# Solvatochromic fluorene-linked nucleoside and DNA as color-changing fluorescent probes for sensing interactions†
†Electronic supplementary information (ESI) available: Additional figures, full experimental section with synthesis and characterization of all compounds, biochemical procedures, spectroscopic measurements, image analysis, computational methods and copies of NMR spectra. See DOI: 10.1039/c6sc02548j


**DOI:** 10.1039/c6sc02548j

**Published:** 2016-06-21

**Authors:** Dmytro Dziuba, Petr Pospíšil, Ján Matyašovský, Jiří Brynda, Dana Nachtigallová, Lubomír Rulíšek, Radek Pohl, Martin Hof, Michal Hocek

**Affiliations:** a Institute of Organic Chemistry and Biochemistry , Czech Academy of Sciences , Gilead & IOCB Research Center , Flemingovo nam. 2 , CZ-16610 Prague 6 , Czech Republic . Email: hocek@uochb.cas.cz; b J. H eyrovský Institute of Physical Chemistry , Czech Academy of Sciences , Dolejskova 3 , CZ-182 23 Prague , Czech Republic; c Department of Organic Chemistry , Faculty of Science , Charles University in Prague , Hlavova 8 , CZ-12843 Prague 2 , Czech Republic

## Abstract

A nucleoside bearing a solvatochromic push–pull fluorene fluorophore (**dC^FL^**) was designed and synthesized by the Sonogashira coupling of alkyne-linked fluorene **8** with 5-iodo-2′-deoxycytidine. The fluorene building block **8** and labeled nucleoside **dC^FL^** exerted bright fluorescence with significant solvatochromic effect providing emission maxima ranging from 421 to 544 nm and high quantum yields even in highly polar solvents, including water. The solvatochromism of **8** was studied by DFT and ADC(2) calculations to show that, depending on the polarity of the solvent, emission either from the planar or the twisted conformation of the excited state can occur. The nucleoside was converted to its triphosphate variant **dC^FL^TP** which was found to be a good substrate for DNA polymerases suitable for the enzymatic synthesis of oligonucleotide or DNA probes by primer extension or PCR. The fluorene-linked DNA can be used as fluorescent probes for DNA–protein (p53) or DNA–lipid interactions, exerting significant color changes visible even to the naked eye. They also appear to be suitable for time-dependent fluorescence shift studies on DNA, yielding information on DNA hydration and dynamics.

## Abstract 2

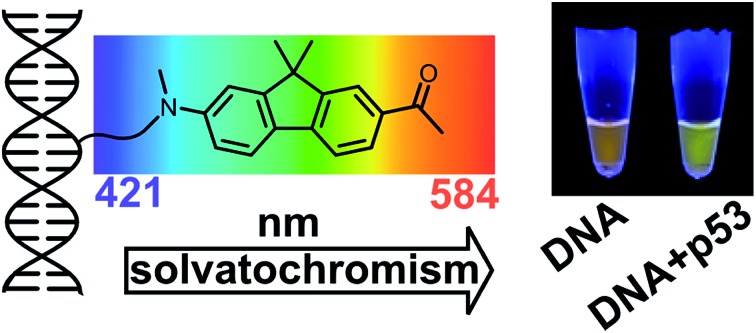
Color-changing fluorescent nucleotide and oligonucleotide probes for studying interactions with other biomolecules were designed and prepared, and perform better than currently known environment-sensitive fluorophores.

## Introduction

Fluorescence spectroscopy in combination with environment-sensitive fluorescent dyes is the method of choice for studying biomolecular interactions.^1^ Fluorophores containing push–pull electron-donating and electron-withdrawing groups attached to a polarizable π-electronic system typically exert solvatochromic properties. They undergo intramolecular charge transfer (ICT) in the excited state resulting in a highly increased dipole moment of the molecule. Fluorescence characteristics of solvatochromic dyes (emission maxima, fluorescence quantum yield, and fluorescence lifetime) strongly depend on the microenvironment mainly due to the dipole–dipole interactions occurring in the excited state.^1^ Due to that, they can sense the local variations of polarity in proteins, lipid membranes and cellular organelles,^1^–^4^ which are often associated with certain biological events. However, to fully reveal the potential of solvatochromic dyes as biophysical probes, they must be site-selectively incorporated into the biomolecules of interest.

**Chart 1:**
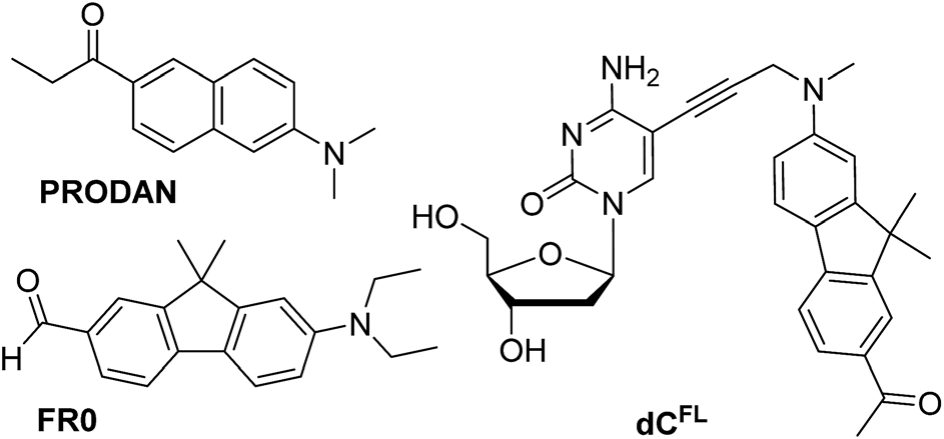
Chemical structures of solvatochromic fluorophores PRODAN and **FR0** described in the literature (ref. 7 and 17*a*, respectively), and nucleoside **dC^FL^** studied in this work.

Diverse environment-sensitive fluorescent nucleoside analogues (FNAs) and their corresponding oligonucleotide probes have been used to study a variety of molecular processes in which nucleic acids (DNA or RNA) are involved. Apart from 2-aminopurine and other FNAs which respond to changes in the microenvironment by changing fluorescence intensity,^5^,^6^ solvatochromic fluorophores provide changes in emission maxima (emission color) as an additional source of information. Particularly, nucleosides with a tethered 6-propionyl-2-(dimethylamino)-naphthalene (PRODAN, Chart 1) moiety^7^ have been used to monitor microenvironmental changes in the major and minor grooves of B and Z-DNA^8^ and for the detection of single nucleotide polymorphism (SNP).^9^ Nucleoside analogs with the nucleobase replaced by solvatochromic fluorophores, such as 4-aminophthalimide^10^ or Nile red,^11^ have been used to probe the polarity within the DNA double helix. A series of isosteric solvatochromic fluorescent analogs of purines and pyrimidines which report on changes in local polarity while not perturbing the stability of nucleic acids have been developed by the Tor group.^12^ Saito and co-workers have developed a wide variety of solvatochromic FNAs based on substituted purines and diverse aza- and/or deaza-analogues of purine bases which probe DNA hybridization.^13^ Ultra-sensitive environment-sensitive nucleosides based on the 3-hydroxychromone fluorophore exhibiting a two-band ratiometric fluorescence response have been reported to probe DNA hybridization and DNA–protein interactions.^14^ Finally, our group has reported solvatochromic nucleoside analogues and their corresponding nucleoside triphosphates (dNTPs) as building blocks for the enzymatic synthesis of DNA probes for hybridization and DNA–protein interactions.^15^

An ideal solvatochromic fluorescence label for DNA should meet the following criteria: (i) it must show high sensitivity to the microenvironment, resulting in a large fluorescence red shift in highly polar media; (ii) it must retain high quantum yield in polar protic solvents, especially water; (iii) it must be sufficiently stable under physiological conditions; (iv) it must be localized at a site on DNA where the changes in local polarity are the most significant; (v) the corresponding 2′-deoxyribonucleoside 5′-*O*-triphosphate (dNTP) must be a substrate for DNA polymerases to allow for the enzymatic preparation of long labelled DNA and to be potentially suitable for labeling DNA *in vivo*. To the best of our knowledge, none of the existing solvatochromic nucleoside analogs meet these criteria. The majority of them were incorporated into DNA only by the phosphoramidite chemical synthesis,^7^–^14^ which significantly limits the size of the oligonucleotide probes which can be prepared. Most of the known solvatochromic nucleosides show only moderate solvatochromic shift, low-to-moderate fluorescence quantum yields in water.^15^,^16^ Some other solvatochromic FNAs are chemically unstable, *e.g.* solvatochromic aminophtalimide nucleosides easily hydrolyze leading to the decomposition of the chromophore.^10^,15b

With the aim to overcome all these limitations, herein we report a new fluorescent 2′-deoxycytidine nucleoside linked to a highly solvatochromic fluorene derivative through a non-conjugated tether, its conversion to dNTP and enzymatic synthesis of color-changing DNA probes for interactions with lipids or proteins. The experimental data are complemented by quantum chemical calculations and molecular dynamics (MD) simulations of the labeled DNA molecule complexed with the p53 protein.

## Results and discussion

### Synthesis

Our interest in designing a solvatochromic nucleoside analogue with improved photophysical properties and chemical stability was stimulated by the fact that a series of new PRODAN-inspired environment-sensitive dyes with superior properties were reported during the last decade.^4^,^17^ We speculated that the fluorene core-based fluorophore **FR0** (Chart 1) reported by Klymchenko and co-workers17*a* would retain its highly favorable environment-sensing properties after tethering to DNA *via* a flexible linker. We proposed nucleoside **dC^FL^** (Chart 1) as the target molecule. Comparing with **FR0**, the fluorophore features a propargyl group grafted to the nitrogen atom to serve as a linker; moreover the reactive formyl group was replaced with acetyl functionality which has less electron-withdrawing properties but is far less reactive. We also note that, in the course of our research, this fluorophore was independently reported as a fluorescent stain for lysosomes.4*b*

**Scheme 1:**
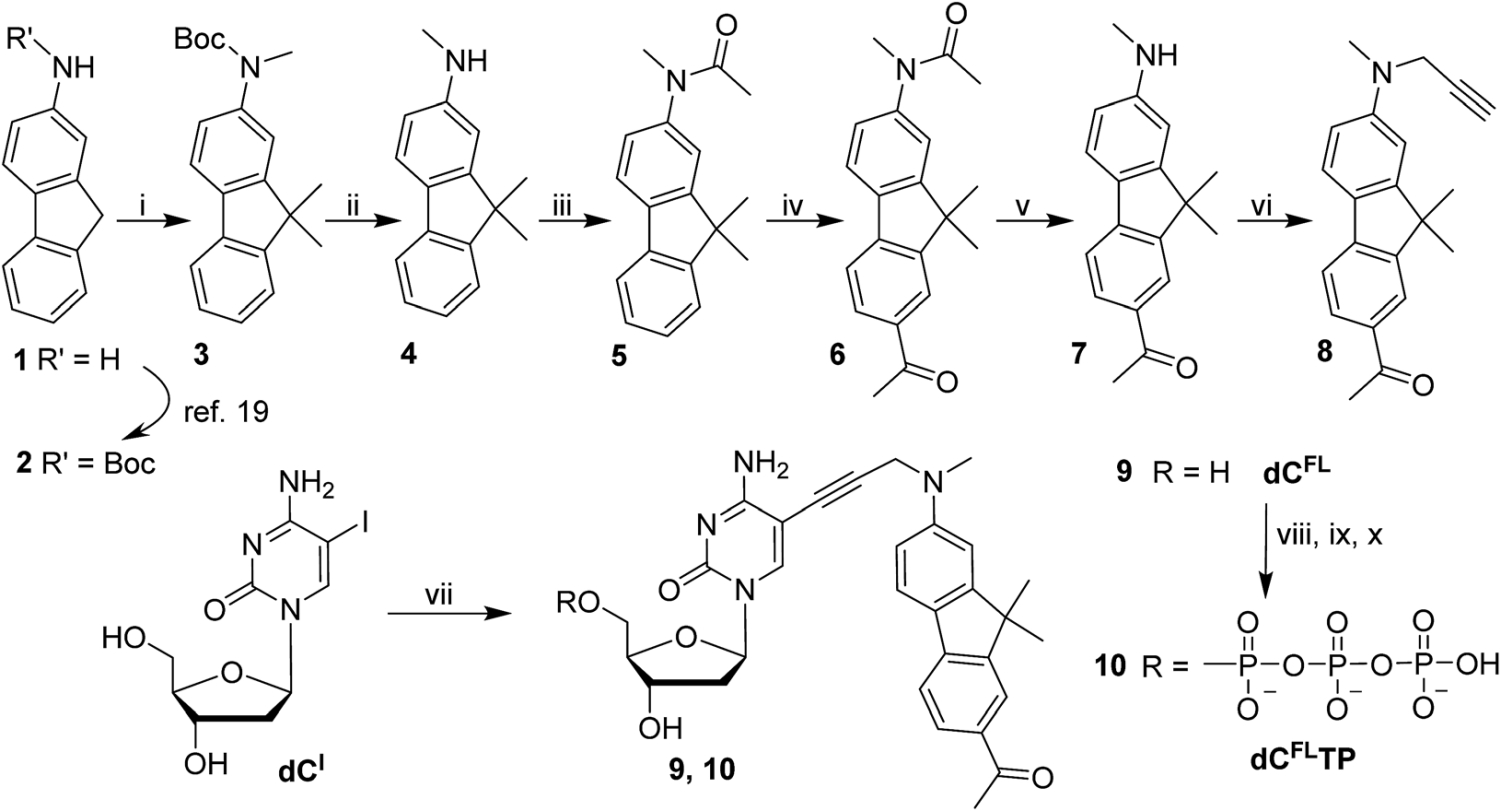
Synthesis of fluorene-labeled 2′-deoxycytidine and 2′-deoxycytidine 5′-*O*-triphosphate derivatives. Reagents and conditions: (i) CH_3_I, *t*-BuOK, THF 0 °C → rt overnight, 83%; (ii) TFA, CH_2_Cl_2_ 0 °C → rt, 1–3 h; (iii) Ac_2_O, THF rt, 1 h, 96% over 2 steps; (iv) AcBr, AlCl_3_, CH_2_Cl_2_ reflux, 4 h, 84%; (v) NaOH, MeOH–H_2_O, 80 °C, 60 h, 81%; (vi) propargyl bromide, K_2_CO_3_, CH_3_CN 70 °C, 24 h, 82%; (vii) **8**, PdCl_2_(PPh_3_)_2_, CuI, NEt_3_, DMF 45 °C, 3 h, 85%; (viii) POCl_3_, PO(OCH_3_)_3_, 0 °C, 4 h: (ix) (*n*-Bu_3_NH)_2_H_2_P_2_O_7_, NBu_3_, DMF, 0 °C, 1 h; (x) 1 M TEAB, 16% from **9**.

The synthesis of the fluorene-labeled nucleoside **dC^FL^** is shown in Scheme 1. At first, we developed a robust and efficient method for the preparation of the alkyne-linked fluorophore **8** building block. It was synthesized from 2-aminofluorene following a convergent strategy with key issues of mono-methylation of the amino group and dimethylation of the fluorene central ring in order to mask undesired reactivity and to red-shift the absorption maximum.^18^ Starting from Boc-protected 2-aminofluorene **2** (ref. 19), all three methyl groups were installed in a one-pot reaction using an excess of methyl iodide and potassium *tert*-butoxide as a base in THF, to give trimethylated compound **3** in 83% yield.

The acid-labile Boc group was then exchanged in two steps to give the more stable acetyl derivative **5** which was used in a Friedel–Crafts acetylation to install the acetyl moiety onto the opposite aromatic ring. The presence of the *N*-acetyl group suppressed possible *ortho*-substitution to give exclusively the 7-acetylated fluorene **6**. Finally, the amide protection on the amine functionality was cleaved by hydrolysis using NaOH in a water–methanol mixture providing secondary amine **7**, which was subsequently propargylated yielding the desired fluorophore-linked terminal alkyne **8**. In this way, the fluorophore building block **8** was prepared from 2-aminofluorene (**1**) in seven synthetic steps with four chromatographic purifications in roughly 42% overall yield, which is significantly easier and more efficient than the alternative route starting from 2-nitro-fluorene published recently.4*b*

The fluorene-linked acetylene (**8**) was then attached to a nucleoside through the Sonogashira cross-coupling with 5-iodo-2′-deoxycytidine (**dC^I^**). The reaction was performed in the presence of PdCl_2_(PPh_3_)_2_ and CuI in DMF and Et_3_N at 45 °C to give the desired fluorescent nucleoside **9** (**dC^FL^**) in 85% yield. Finally, to obtain the substrate for polymerase incorporation into DNA, the nucleoside was phosphorylated under Ludwig's conditions^20^ giving the corresponding solvatochromic fluorene-tethered dCTP analogue **10** (**dC^FL^TP**) in 16% yield.

### Photophysical properties

**Fig. 1:**
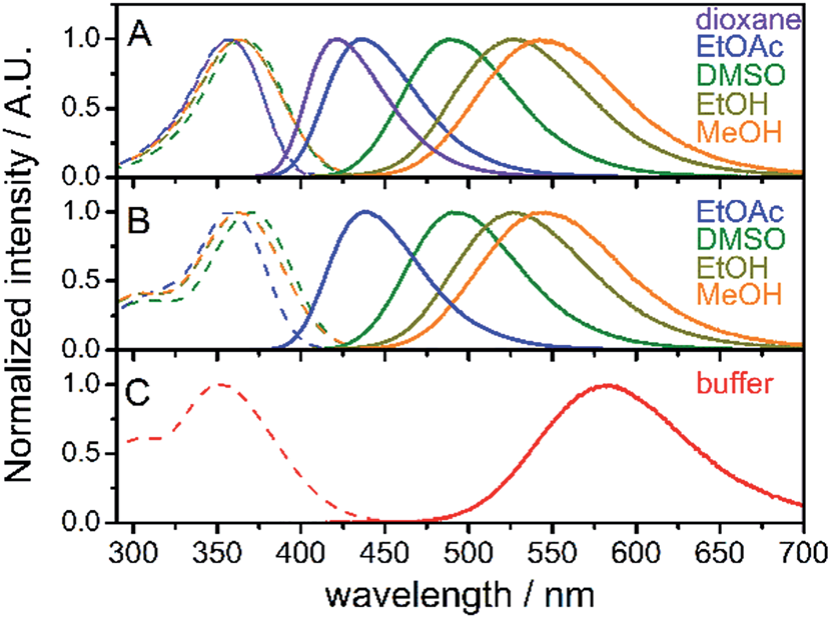
Normalized absorption (dashed lines) and fluorescence (solid lines) of compounds **8** (A), **dC^FL^** (B) and **dC^FL^TP** (C) in solvents of different polarity; buffer: 10 mM sodium phosphate buffer, pH 7.4.

Absorption and steady-state fluorescence spectra of fluorene fluorophore **8** were studied in a set of solvents of varying polarity and compared with those of nucleoside **dC^FL^** and triphosphate **dC^FL^TP**; the results are summarized in Fig. 1 and Table 1. The fluorophore exhibited relatively high solvent-dependent absorption coefficients (up to 30 700 M^–1^ cm^–1^). The absorption spectra of fluorene **8** centered within a narrow range of 357–366 nm in these solvents. The hypsochromic shift in the absorption of **8** compared to the parent compound **FR0** (390–399 nm)17*a* can be explained by the weaker electron-withdrawing properties of the acetyl group over the formyl group, which rendered a lower excited state dipole moment of the molecule. A strong solvent-dependent variation of emission maxima was observed for **8**. The positions of the emission maxima ranged from 421 nm in dioxane to 544 nm in methanol. Hence, emission colors of the solvatochromic fluorene are similar to those reported for **FR0**17*a* and cover a significant part of the visible spectrum, from deep-blue to reddish orange (Fig. S1a†). More importantly, high fluorescence quantum yields were observed for the whole range of studied solvents, including protic methanol which readily quenches the emission of solvatochromic dyes.16b,^21^ Nucleoside **dC^FL^** exhibited similar spectroscopic properties (Table 1, Fig. 1B). The positions of absorption and emission bands were nearly identical to **8**, indicating that the tethering of the nucleoside *via* the non-conjugate propargyl linker did not change the electronic properties of the fluorophore. At the same time, fluorescence quantum yields were 0.06–0.10 lower than those of **8**, which can be explained by the higher number of rotatable bonds in the nucleoside and therefore its higher conformational mobility. Nevertheless, the nucleoside retained relatively high quantum yields of fluorescence even in polar protic solvents (0.64 and 0.57 for ethanol and methanol, respectively). The nucleoside triphosphate **dC^FL^TP** dissolved in aqueous buffer exhibited an absorption band of the fluorophore centered at 352 nm and the reddest emission spectrum centered at 584 nm, resulting in a large Stokes shift of 232 nm (Fig. 1C). To our satisfaction, despite this large solvatochromic shift the fluorophore did not suffer from excessive quenching by water. The nucleoside triphosphate exhibited high fluorescence quantum yield (0.27), which to the best of our knowledge is one of the highest values ever exhibited by solvatochromic nucleoside analogs.^22^ The Stokes shifts of all the measured compounds ranged from 4400 cm^–1^ for **8** in 1,4-dioxane to 11 400 cm^–1^ for **dC^FL^TP** in aqueous buffer. Interestingly, the Stokes shift of the fluorophore perfectly correlates with the empirical solvent polarity scale Et(30)^23^ (Fig. S1b†). Altogether, these experiments show that the solvatochromic fluorene fluorophore has good potential for DNA labeling and for probing DNA interactions.

**Table 1:** Spectroscopic properties of the solvatochromic fluorene (**8**) and of the corresponding labeled nucleoside (**dC^FL^**) and nucleoside triphosphate (**dC^FL^TP**) derivatives

Compd	Solvent	*ε* [10^3^ M^–1^ cm^–1^]	*λ*_abs_ ^*a*^ [nm]	*λ*_em_ ^*b*^ [nm]	*Φ*_f_ ^*c*^
**8**	1,4-Dioxane	30.7	357	421	0.58
	CH_2_Cl_2_	n.d.^*d*^	362	458	0.86
	EtOAc	n.d.	357	436	0.67
	DMF	n.d.	364	476	0.81
	DMSO	28.3	366	490	0.85
	Acetonitrile	n.d.	358	479	0.76
	Acetone	n.d.	359	469	0.75
	*n*-BuOH	n.d.	366	519	0.76
	EtOH	27.5	364	528	0.70
	MeOH	n.d.	363	544	0.63
**dC^FL^**	EtOAc	n.d.	357	438	0.59
	DMSO	n.d.	370	491	0.75
	DMF	n.d.	366	479	0.74
	EtOH	n.d.	365	528	0.64
	MeOH	n.d.	364	544	0.57
**dC^FL^TP**	Buffer^*e*^	n.d.	352	584	0.27

^*a*^Position of the absorption maximum, ±1 nm.

^*b*^Position of the emission maximum, ±2 nm.

^*c*^Quantum yield of fluorescence measured using quinine sulfate in 0.5 M H_2_SO_4_ (*Φ*_f_ = 0.55 at 25 °C) as a standard.

^*d*^n.d. = not determined.

^*e*^10 mM sodium phosphate buffer, pH 7.4.

### Quantum chemical calculations of the emission spectra

Further insight into the origin of the color-changing behaviour of the studied nucleoside can be obtained from quantum chemical calculations. An excellent starting point for our computational study is the theoretical description of the emitting state of the structurally related PRODAN molecule (Chart 1) which identified two distinct S_1_ equilibrium geometries – planar or twisted^24^,^25^ and calibrated a computational TD-DFT (B3-LYP) and resolution-of-identity algebraic diagrammatic construction through second order RI-ADC(2) methods (see ESI† for Computational details).

**Fig. 2:**
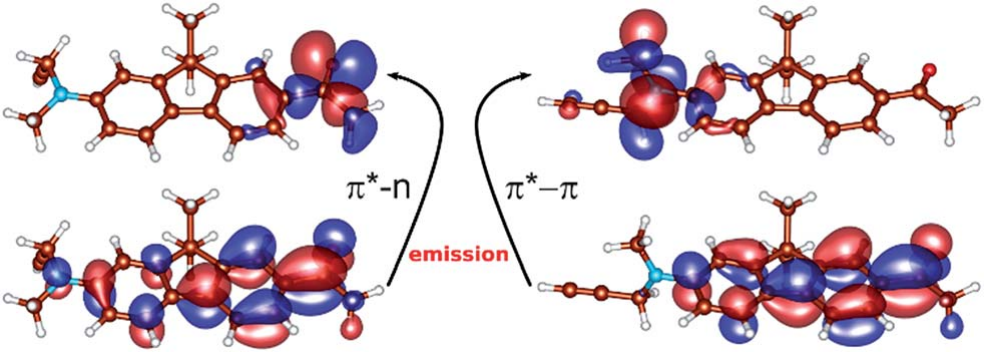
The orbitals involved in the emission of **S_1_p** (left): top – lone pair orbital localized on the oxygen of the carbonyl group; bottom – the delocalized antibonding π* orbital, and **S_1_t** (right): top – the orbital of the π-character localized on the nitrogen of the twisted substituted amino-group, bottom-delocalized π* orbital. Molecular orbitals were generated by the CHARMOL program written by J. Chalupský.

The results of the calculations of the vertical absorption spectra at the ground-state equilibrium geometry of **8** are presented in Table 2, both in the gas-phase and in the solvent (modeled by polarized continuum). Both TD-DFT and ADC(2) methods clearly elucidated the nature of the S_1_ and S_2_ excited states. One of the two excitations is of π → π* character, where the two orbitals involved in the excitation are delocalized over the polycyclic aromatic system (Fig. 2). This is responsible for an intense absorption peak at ∼365 nm observed experimentally in alcohols and computed at 375 nm employing the ADC(2) method and the *ε*_r_ = 80 polarized continuum (*c.f.*Tables 1 and 2). The second excited state (mostly S_2_) is of the n → π* character where the lone pair orbital is localized on the oxygen of the CO group (Fig. 2), and appears as a low-intensity peak at 310 nm [ADC(2), *ε*_r_ = 80].

**Table 2:** Absorption energies (*E*_abs_) and oscillator strengths (*f*) calculated for **8**

Method	*ε*_r_ ^*b*^	*E*_abs_/*λ*_max_ (eV/nm)	Character	*f*
			**S**_**1**_	
TD-DFT/B3LYP^*a*^	1(g.p)	3.433/361	π → π*	0.70
ADC(2)^*a*^	1	3.656/339	n → π*	2 × 10^–3^
	80	3.310/375	π → π*	0.87

			**S**_**2**_	
TD-DFT/B3LYP^*a*^	1(g.p)	3.716/334	n → π*	9 × 10^–5^
ADC(2)^*a*^	1	3.684/337	π → π*	0.85
	80	4.004/310	n → π*	0.02

^*a*^Employing def2-SVP basis set.

^*b*^Dielectric constant.

Concerning the fluorescence properties of **8**, the TD-DFT (B3-LYP)/def2-TZVP gas-phase S_1_ geometry optimizations resulted in two distinct excited state minima (Fig. 2). Their emissive properties are listed in Table 3. The two structures differ mainly by the rotation of the substituted amino-group with respect to the plane of the aromatic carbon rings, resulting in semi-planar (**S_1_p**) and twisted (**S_1_t**) structures, respectively. In the semi-planar (**S_1_p**) structure, the S_1_ excited state is of n → π* character (*vide supra* and Fig. 2), while the twisted structure (**S_1_t**) is characterized by the π → π* S_1_ transition with a charge transfer character (Fig. 2). The single-point polarized continuum (*ε*_r_ = 2.2, 38.6 and 80 to model 1,4-dioxane, acetonitrile and water environments) ADC(2) calculations at the respective TD-DFT (B3-LYP/def2-TZVP) gas-phase optimized geometries were carried out to elucidate the origin of the observed Stokes shifts of **8**. With the increasing polarity of the environment the relative stability of the less polar **S_1_p** state, characterized by dipole moment *μ* = 2.1 D, decreases with respect to the **S_1_t** (*μ* = 22.5 D). While the **S_1_p** state is stabilized by 0.67 eV over the **S_1_t** state in dioxane, this difference is only 0.19 eV in water. The calculated emission energies of both **S_1_p** and **S_1_t** are shown in Table 3. The increasing polarity of the solvent results in a blue shift of the less polar **S_1_p** state, from 467 nm (2.63 eV) in *ε*_r_ = 2 to 450 nm (2.73 eV) in *ε*_r_ = 80. The opposite trend has been observed for the more polar **S_1_t** state, *i.e.* a red shift from 427 nm (2.90 eV) in *ε*_r_ = 2 to 583 nm (2.13 eV) *ε*_r_ = 80, in accordance with the experimental observations. Thus, the computed values provide us with a consistent picture of the nature of the solvatochromism observed in the emission spectra. The results indicate that both states may play a role in the emission depending on the polarity of the solvent. In less polar solvent, the emission from the **S_1_p** geometry might occur whereas in polar solvents the twisted conformation **S_1_t** is the likely representation of the emitting state.

**Table 3:** Emission energies (*E*_emiss_) and relative energies (*E*_rel_) calculated^*a*^ for the S_1_ state in the semi-planar (**S_1_p**) and twisted (**S_1_t**) geometries of **8** in different environments

*ε*_r_ ^*b*^	*E*_rel_ ^*c*^ (eV)	*E*_emiss_/*λ*_max_ (eV/nm) **S_1_p**^*d*^	*E*_emiss_/*λ*_max_ (eV/nm) **S_1_t**^*e*^	Experiment *E*_emiss_/*λ*_max_ (eV/nm)
1.0	0.92	2.627/472	3.200/387	
2.2	0.67	2.654/467	2.903/427	2.945/421
38.6	0.26	2.687/461	2.204/562	2.643/469
80	0.19	2.730/454	2.126/583	2.123/584

^*a*^Calculated by ADC(2) employing the def2-TZVP basis set.

^*b*^Dielectric constant.

^*c*^The energy difference *E*_rel_ = *E*(**S_1_p**) – *E*(**S_1_t**).

^*d*^ **S_1_p**: *r*(CO) = 1.297 Å, deviation from the planarity 12 and 8 deg, dipole moment *μ* = 2.1 D.

^*e*^ **S_1_t**: *r*(CO) = 1.238 Å, deviation from the planarity 89 and 91 deg, dipole moment *μ* = 22.5 D.

Finally, it can be mentioned that an attempt has been made to use the qualitative model of the protein–**DNA^FL^** complex described in this study (*vide infra*) and calculate emission spectra in the context of DNA-bound fluorene interacting with the protein. However, the potentially quantitatively accurate ADC(2) calculations turned out to be beyond current computational feasibility and TD-DFT calculations, with several explicit protein residues included which did not provide data of sufficient accuracy that would go significantly beyond the predictions based on the polarity of the environment modelled by the implicit solvation model (*vide supra*).

### Polymerase incorporation of fluorene-linked nucleotide

**Fig. 3:**
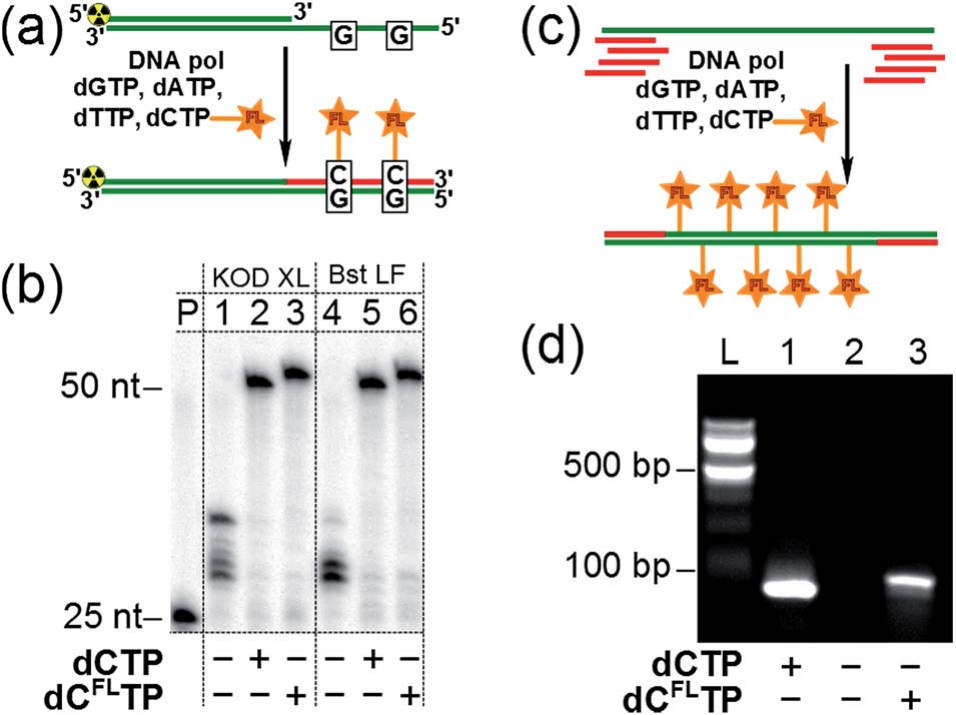
(a) Synthesis of fluorene-labeled double-stranded DNA by PEX with modified **dC^FL^TP** and three remaining natural dNTPs as substrates. (b) PAGE analysis of the PEX products made by KOD XL DNA polymerase (lanes 1–3) and Bst DNA polymerase large fragment (lanes 4–6). P: primer. Negative control: dTTP, dCTP, dGTP (lanes 1, 4). Positive control: all four natural dNTPs (lanes 2, 5). Modified dNTP: **dC^FL^TP**, dATP, dGTP, dTTP (lanes 3, 6). (c) Synthesis of multiply-labeled double stranded DNA by PCR amplification of a 98 bp template using **dC^FL^TP** and three remaining natural dNTPs: the total number of modified residues in the product was 34. (d) Agarose gel electrophoresis analysis of PCR with KOD XL polymerase and **dC^FL^TP**. L: 100 bp ladder. Positive control: all four natural dNTPs (lane 1). Negative control: dTTP, dATP, dGTP (lane 2). Modified dNTP: **dC^FL^TP**, dATP, dGTP, dTTP (lane 3).

Subsequently, we investigated whether the modified triphosphate **dC^FL^TP** can be used for the polymerase synthesis^26^ of DNA with a grafted solvatochromic fluorene. We tested **dC^FL^TP** in a primer extension (PEX) reaction using a 50 nt template and 25 nt primer designed so that the product would contain two deoxycytidines (Fig. 3a). When the PEX was performed in the presence of KOD XL DNA polymerase or Bst DNA polymerase (large fragment), polyacrylamide gel electrophoresis (PAGE) (Fig. 3b) showed a clean formation of the full-length primer extension products when **dC^FL^TP** was used in combination with the three remaining natural dNTPs (lanes 3, 6). These products had similar mobility as those obtained by PEX with natural dNTPs (positive control, lanes 2, 5). Other popular DNA polymerases, namely *Vent*(*exo*-), *Pwo*, *Phusion*, 9°N_m_, DyNAzyme II, were unable to efficiently use **dC^FL^TP** as a substrate (data not shown). Correct incorporation of **dC^FL^TP** into DNA by KOD XL polymerase was also confirmed by PEX with a biotinylated template followed by magnetic separation with streptavidin magnetic beads followed by MALDI-TOF analysis (Fig. S2†).

We also found out that KOD XL is able to use **dC^FL^TP** in PCR amplification yielding the labeled 98 bp-long double-stranded DNA containing as many as 34 fluorene-modified deoxycytidine residues (Fig. 3c and d). Finally, we showed that the fluorene-labeled DNA can be purified from the unreacted fluorene-labeled triphosphate using a widely used method based on absorption of DNA on a silica-gel membrane in the presence of chaotropic salts.^27^ A primed DNA template was incubated with the modified and three natural dNTPs either in the presence or in the absence of the polymerase and then DNA was isolated from both samples using one of the commercially available DNA purification kits. The incorporation of the fluorene moiety into DNA was unambiguously demonstrated for the polymerase-positive sample by UV-vis absorption and steady-state fluorescence spectroscopy (Fig. S3†). On the contrary, the fluorophore was not detected in the polymerase-negative sample, indicating that the fluorene-modified nucleoside triphosphate do not interact nonspecifically with DNA *via* intercalation and can by efficiently removed using standard techniques.

### DNA–protein interactions

Having a robust and facile method for the preparation of labelled oligonucleotides and DNA, we proceeded to the study of their properties. To produce 50-bp fluorene-labelled dsDNA, we used semi-preparative PEX in the presence of KOD XL DNA polymerase (Scheme S1†). This DNA (referred to here as **DNA^FL^**) bears two tethered solvatochromic fluorene moieties and contains the consensus recognition site of human transcription factor p53 from our previous studies.^28^ Circular dichroism (CD) spectra showed that the attachment of the fluorophores did not significantly alter the B-DNA conformation of the DNA (Fig. S4†). The fluorescence spectrum of **DNA^FL^** was centred at 581 nm, which was only a 3 nm blue-shift compared to the triphosphate in water. Such a small blue shift between the dNTP in buffer and DNA indicates only a minor shielding effect of DNA or small dielectric differences between water in the bulk and the hydration shell of DNA in the vicinity of the major groove, where the probe is located. From the calibration curve on Fig. S1b† one can estimate that the media surrounding the fluorophore can be characterized by Et(30) ∼ 60 kcal mol^–1^. This value is higher than that which was concluded in previous studies using other solvatochromic nucleosides12c,^29^ and is in line with the observation that the spectroscopically measured values of polarity of the major groove depend on the nucleoside probe and linker used.12*c*

**Fig. 4:**
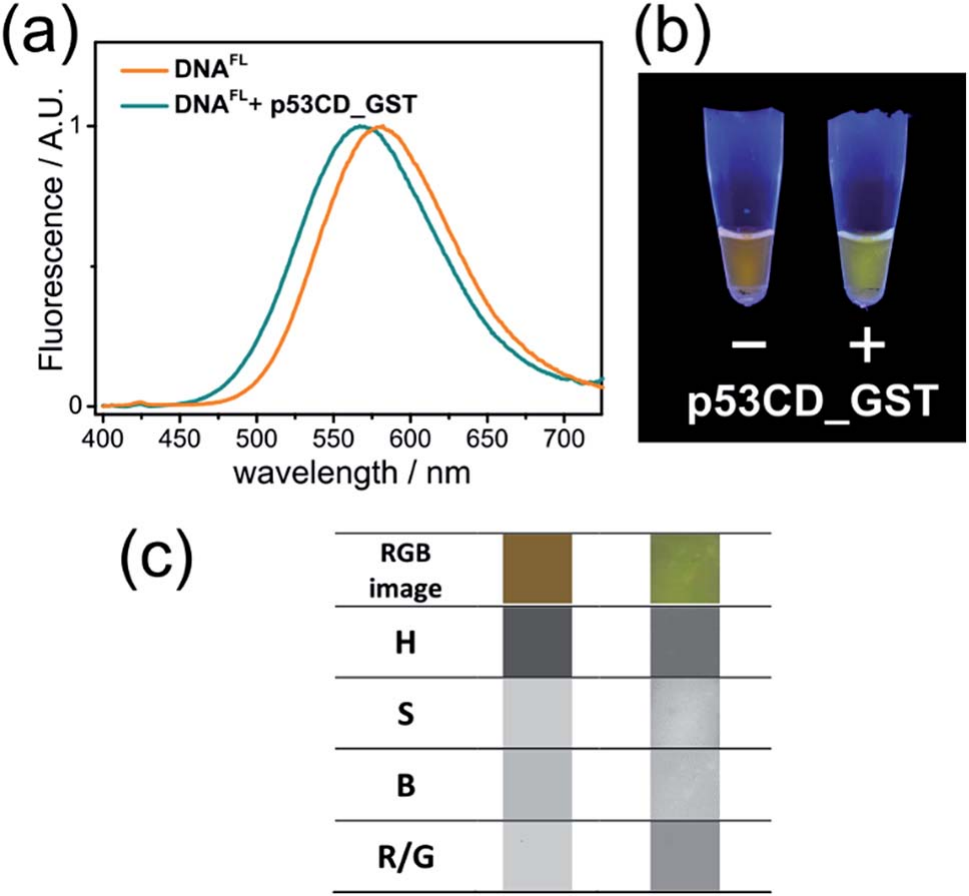
(a) Normalized fluorescence spectra (*λ*_exc_ = 365 nm) and (b) photography of UV-illuminated solutions of **DNA^FL^** with and without **p53CD_GST**; (c) image analysis of photography of **DNA^FL^** with and without **p53CD_GST**. From the top to the bottom row: RGB segment from the photography, hue (H), saturation (S), brightness (B) and red/green ratio (R/G).

To explore the applicability of the solvatochromic fluorene nucleoside as a colour-changing reporter of biomolecular interactions, we studied the influence of protein binding on the fluorescence properties of **DNA^FL^**. In this study we used the core domain of the human transcription factor p53 with a GST tag (**p53CD_GST**) expressed in bacteria as a model DNA-binding protein.^30^ First, we examined whether the tethering of the bulk hydrophobic fluorophore within the recognition site would not disrupt the ability of the DNA to be recognized by the protein. An electrophoretic mobility shift assay (EMSA) with a radioactively labeled **DNA^FL^** on PAGE under native conditions showed no significant difference in the binding of **p53CD_GST** between the labeled **DNA^FL^** and non-modified DNA control (Fig. S5†). When fluorescence spectra of **DNA^FL^** were measured either with or without **p53CD_GST**, the protein-bound **DNA^FL^** showed a blue shift in the fluorescence spectrum. The emission maximum shifted from 581 nm to 567 nm (Fig. 4a) indicating partial screening of the fluorophore within the DNA–protein complex. Nevertheless, the value of the emission maximum was far from the values obtained for **8** in non-polar solvents, indicating that the media surrounding the fluorophore remained fully hydrated. Notably, the hypsochromic shift obtained in this experiment was *ca.* 1.8-fold larger than that reported by Saito and co-workers who used another PRODAN-like nucleoside to probe DNA binding to the Klenow fragment of DNA polymerase I from *Escherichia coli*.^29^ To the best of our knowledge, this is the first example where a change of color in a labelled DNA probe upon binding to a protein was distinguishable by the naked eye (Fig. 4b).

Color-changing fluorescent probes in combination with digital RGB cameras and image-processing software are becoming important tools for practical implementation of the fluorescence sensing technology.^31^ We estimated the applicability of our probes in this context. Images of fluorescent solutions were taken with a digital colour camera and analysed using the image-processing package ImageJ as described in the literature.31*c* To our satisfaction, the changes of emission colour were suitable for quantification (Fig. S6–S8 and ESI† for details). Further, image analysis of the **DNA^FL^** and **DNA^FL^**–**p53CD_GST** solutions shown in Fig. 4b was performed in terms of ratiometric and hue quantification.31*c* The changes in hue (H) and red/green (R/G) ratio (Fig. 4c) reflecting that different microenvironment of the probe can be distinguished. Notably, the change of ratiometric signal (R/G) were superior compared to the difference in hue.

**Fig. 5:**
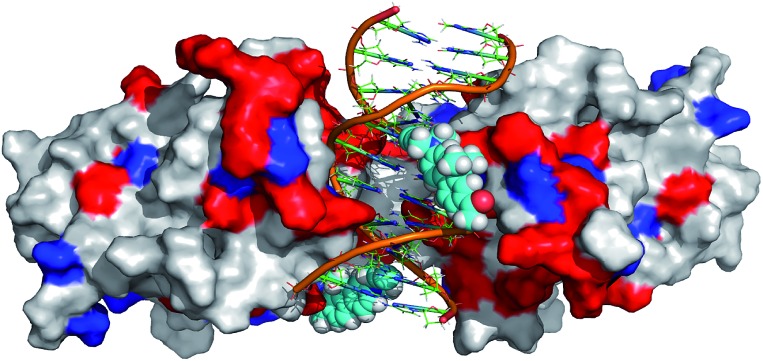
Modeled structure of p53 bound to the labelled DNA probe (**DNA^FL^**). Protein p53 is depicted as a solvent accessible surface, colored white for hydrophobic and polar amino acids (AA), blue for negatively charged AA and red for positively charged AA. The DNA duplex is depicted in cartoon representation and fluorophores as vdW spheres.

In order to further rationalize the experimentally observed color change upon interaction of the labelled DNA molecule with the protein and provide a missing link between quantum chemical calculations and experiment, an attempt was made to predict the three-dimensional structure of the complex. To this aim, the available crystal structure of the p53 + DNA complex (PDBID 3EXJ) was used as a template and modified using the YASARA modelling package.^32^ The fluorene molecules were covalently linked to the corresponding nucleobases in the DNA. Next, hydrogen atoms (missing in the crystal structure) were added to the protein into their standard positions with the protonation states corresponding to the neutral pH and their position were subsequently optimized. The whole structure was immersed into the solvent box (periodic boundary conditions, PBC). The system was electro-neutralized by placing the appropriate number of Na^+^ and Cl^–^ ions corresponding to their 0.15 M concentration. The AMBER ff03 force field^33^ and parameter set were used for the protein and DNA. Ligand **8** was optimized in a vacuum and partial charges on its atoms were obtained by a restrained fit to the electrostatic potential (RESP) at the AM1BCC level^34^ whereas standard *gaff* parameters were used for bonded and van der Waals terms of **8**. After initial minimization, the simulated annealing protocol, *i.e.* repeated cycles of molecular dynamics under the PBC, with YASARA defaults^32^ was applied and the resulting model was subjected to final minimization. The full structure is given in the ESI† whereas the fluorene environment is displayed in Fig. 5.

Though such a simplified model does not allow one to quantify the polarity change, the modeled structure (Fig. 5) clearly shows that the fluorene labels at least partly interact with the protein. Apparently, this interaction changes the polarity of the environment around the labels from highly polar water to a less polar protein and this change is significant enough to shift the emission maxima of the fluorophores by 14 nm resulting in a naked-eye-visible color change from orange to yellow.

### DNA–lipid interactions

**Fig. 6:**
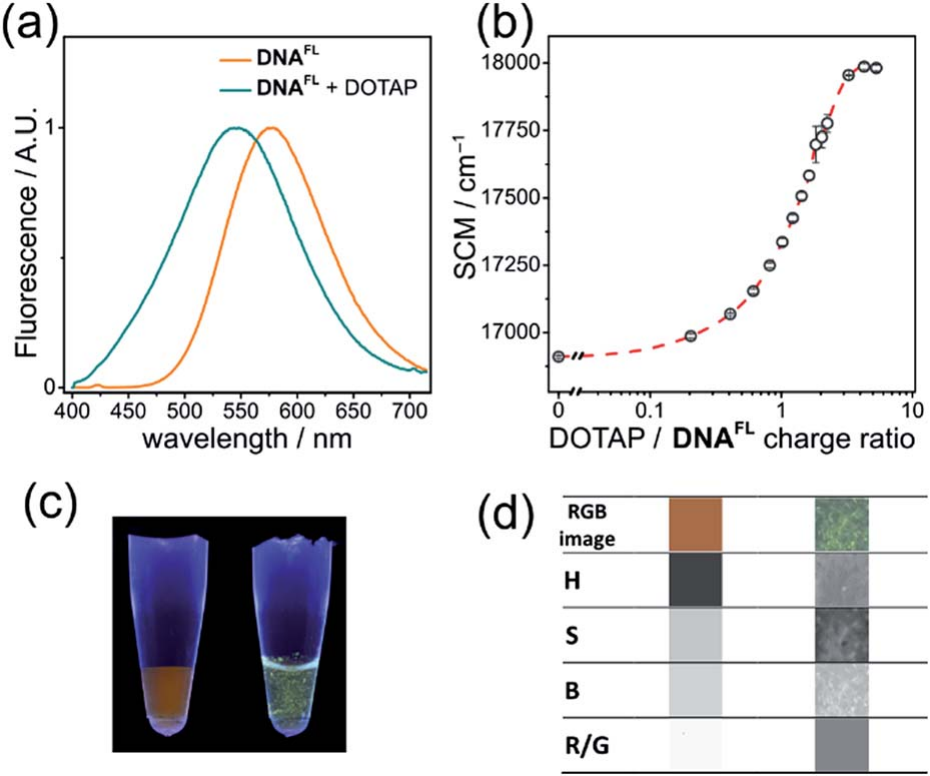
**DNA^FL^** changes emission color upon formation of lipoplexes with DOTAP. (a) Normalized fluorescence spectra (*λ*_ex_ = 365 nm) of **DNA^FL^** before (orange) and after (cyan) addition of DOTAP SUVs; (b) dependence of the **DNA^FL^** spectral center of mass (SCM) on the DOTAP/DNA charge ratio; data points show the mean value ± s.e.m. (*n* = 3). (c) Photography and (d) image analysis of **DNA^FL^** with and without DOTAP. From the top to the bottom row: RGB segment of the photography, hue (H), saturation (S), brightness (B) and red/green ratio (R/G).

Cationic lipids are important nucleic acid-transferring reagents widely used in cell biology and in gene therapy.^35^ Therefore, we decided to examine whether the formation of lipoplexes (compact complexes of DNA and lipids) can be monitored by the fluorescence of the solvatochromic fluorene fluorophore in order to study the influence of cationic lipid DOTAP on the fluorescence of **DNA^FL^**. We observed a significant blue shift in the emission maximum accomplished by a change in the shape of the spectrum upon addition of DOTAP small unilamellar vesicles to DNA. The observed 33 nm blue shift was easily observable by the naked eye at sub-micromolar concentrations of DNA (Fig. 6a, c) and it was even more significant than in the case of protein binding (**p53CD_GST**).

The plot of the spectral center of mass (SCM) *versus* DOTAP/**DNA^FL^** charge ratio had a sigmoidal shape (Fig. 6b) with a transition at charge ratio ∼1, in line with our previous observations.28*b* Further, we analyzed the RGB images of UV-illuminated **DNA^FL^** and DOTAP–**DNA^FL^** samples in the same way as described above for DNA–protein interactions (Fig. 6c and d). Again, one can notice apparent changes in H and R/G ratio upon addition of DOTAP. Altogether, these experiments prove the utility of **dC^FL^** as a fluorescent color-changing probe for studies of DNA interactions *in vitro*.

### Time-dependent fluorescence shift (TDFS) assay

In contradiction to the above shown steady-state data, the time-dependent fluorescence shift assay provides direct and simultaneous information on hydration and mobility of the intimate dye's solvent shell.2*c* To that end, we performed time-resolved emission spectroscopy (TRES) of **dC^FL^** and thus monitored the so-called solvent relaxation process at the corresponding position within the DNA molecule. Previous investigations of DNA solvation dynamics were performed with probes placed inside the duplex-DNA either by covalent attachment or by non-covalent minor groove binding.^36^ The characterized dynamics in DNA were found to be nonexponential and extended into subpicosecond to nanoseconds timescales, best described by a power-law relaxation.^37^ There is no unified explanation of such dispersed dynamics primarily because of the complicated coupling between motions of components localized in the closest (<1 nm) vicinity of the probe: water molecules, segments of the DNA molecule as well as cations. However, for an interpretation one should keep in mind that the solvent relaxation response of bulk water occurs on a sub-picosecond time scale^38^ and slower responses (*i.e.* on the ps and ns time-scale) have to be attributed to DNA dynamics.^39^

**Fig. 7:**
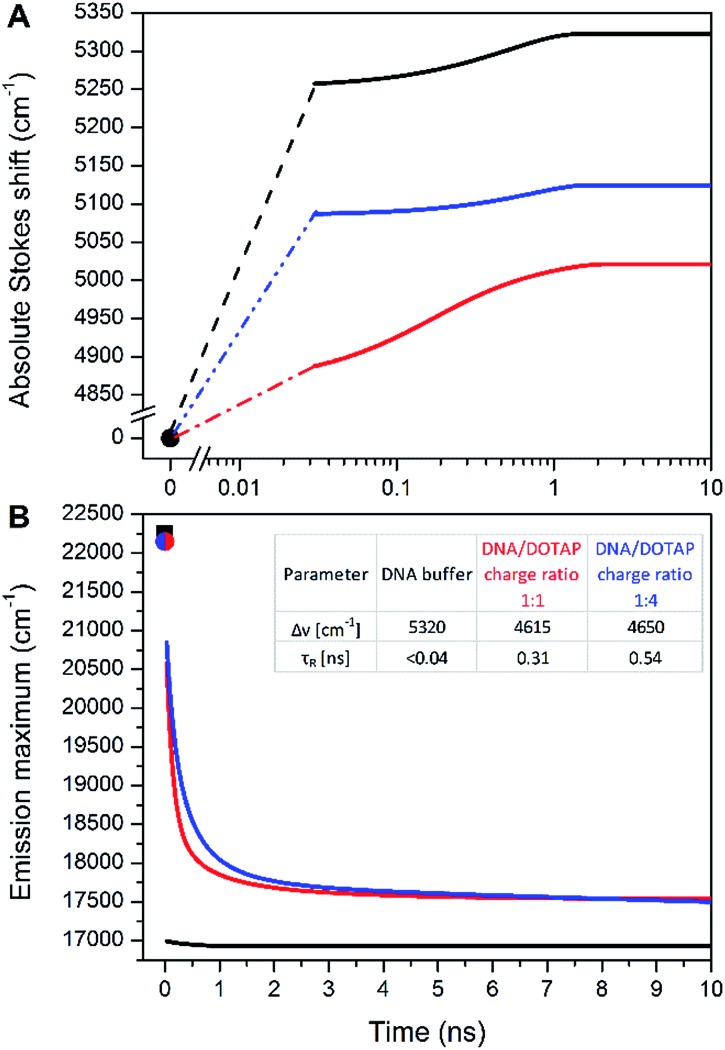
(A) Time evolution of the TRES maxima *v*(*t*) for **DNA^FL^** in buffer (black), in a mixture of buffer and glycerol (ratio 1 : 1 – red), in buffer with p53CD_GST (blue). (B) Time evolution of the TRES maximum for **DNA^FL^** in buffer (black), **DNA^FL^**/DOTAP 1 : 1 charge ratio (red) and **DNA^FL^**/DOTAP 1 : 4 charge ratio (blue). *v*pem(0) is estimated at 22 250 cm^–1^ for DNA (black square) and 22 150 for **DNA^FL^**/DOTAP (red and blue point).

In this work, we measured temporal evolution of the emission maximum of the labelled **DNA^FL^** with **dC^FL^** exposed to the major groove of DNA in several microenvironments. The experimentally observed transient Stokes for **DNA^FL^** in buffer, buffer–glycerol mixture (1 : 1) and **DNA^FL^**–**p53CD_GST** complex are 70 cm^–1^, 130 cm^–1^, and 40 cm^–1^, respectively. These values are negligible compared to the total amount of Stokes shift of 5320 cm^–1^, 5020 cm^–1^, and 5120 cm^–1^, respectively, obtained by the “time-zero” estimation (Fig. 7). These findings indicate that the nano-vicinity of the probe is different to the probe in plain water (estimated total amount of Stokes shift of **dC^FL^TP** is about 5600 cm^–1^), but highly hydrated. The significantly smaller Stokes shift for the **DNA^FL^**–**p53CD_GST** complex indicates a less hydrated probe environment when compared to the free DNA molecule. As practically the entire solvation dynamics are occurring on a time-scale faster than that of the resolution of the experiment (*i.e.* faster than 40 ps), the ensemble solvent dynamics are due to fast segmental motions of the DNA with possibly some contribution of bulk water. These observations are comparable with literature reported dynamic Stokes shifts: independent of chromophore (*i.e.* coumarin 102, 4′,6-diamidino-2-phenylindole, Hoechst 33258 and 2-hydroxy-7-nitro-fluorene (HNF)) and way of labelling (nucleobase replacement or non-covalent minor groove-binding), the largest part of the transient Stokes shift occurs on a time scale faster than 40 ps.^37^,^40^ However, in all these cases, except for HNF, a minor, but significant nanosecond component is present.^36^,^41^

A significantly different picture was observed for **DNA^FL^** incorporated into the **DNA^FL^**/DOTAP lipoplexes. The total amount of the Stokes shift decreased to 4650 cm^–1^. Even more strikingly, the experimentally observed transient Stokes shifts increased to 65% and 72% of the total amount of the Stokes shifts in the cases of 1 : 1 **DNA^FL^**/DOTAP charge ratio and 1 : 4 **DNA^FL^**/DOTAP charge ratio, respectively (Fig. 7). In this case, almost all solvation dynamics occurred on the nanosecond time scale. Upon addition of DOTAP to **DNA^FL^** accompanied by lipoplex formation between **DNA^FL^** and DOTAP, the nano-vicinity of **DNA^FL^** became significantly less hydrated compared to **DNA^FL^** alone in the buffer and the microenvironment mobility decreased dramatically. Addition of more DOTAP to **DNA^FL^/**DOTAP lipoplexes resulted in a further decrease of the slower relaxation time (*τ*_R_) while the hydration stayed at almost the same level. This observation suggests that the lipoplex structure is getting more compact upon addition of the DOTAP, which is in line with previously reported results, where the experiments with the addition of DOTAP resulted in more compressed lipoplexes and growth in their size.28*b* As the time-scale of the TDFS is comparable to probes located at the external interface of a lipid,^42^ we speculate that the **dC^FL^** dye incorporated in the **DNA^FL^** molecule is probing mainly the hydrated positively charged DOTAP head-groups which are in close contact with **DNA^FL^** incorporated into the **DNA^FL^**/DOTAP lipoplexes. We are now performing more experiments to explain the **DNA^FL^**/DOTAP dynamics in detail.

Notably, such a nanosecond TDFS has not been described in a DNA system. Together with the large total Stokes shift, we believe that **dC^FL^** has good potential as a fluorescence probe in applications in the TDFS solvation studies of DNA. Furthermore, lipoplex investigation is of interest for the gene transfer to the cells. Studying **DNA^FL^** lipoplexes could reveal not only DNA dynamics themselves but also the processes involved in DNA delivery into cells.^43^

## Conclusions

To conclude, we developed a new solvatochromic nucleoside (**dC^FL^**) featuring a unique combination of properties: (i) its triphosphate (**dC^FL^TP**) is recognized as a substrate by DNA polymerases in primer extension and PCR; (ii) it does not suffer from hydrolytic instability in aqueous solution; (iii) it exhibits exceptional solvatochromicity, namely the shift in emission band from 421 nm in dioxane to 584 nm in water, covering nearly the whole visible spectrum; (iv) the dye retains a relatively high quantum yield of fluorescence in water. The changes in emission color of the labeled DNA upon interactions with DNA-binding protein p53 and lipid are quite pronounced and detectable not only by fluorescence spectroscopy but also by digital photography with image analysis and by naked eye inspection. To the best of our knowledge, this is the first naked-eye-visible sensor to study DNA protein interactions and the push–pull fluorene-based solvatochromic fluorophore performs better than environment-sensitive fluorophores previously used for the labeling of nucleic acids.^12^–^16^

The physico-chemical origins of the observed solvatochromism were rationalized by DFT and ADC(2) calculations correlated with the computational model of the DNA + protein complex which show that, depending on the polarity of the solvent, emission either from the planar or twisted conformation of the excited state occurs resulting in a change of the emission wavelength. Finally, the modified nucleoside was shown to be suitable for probing site-specific dynamics and hydration of DNA using TDFS measurements. The combination of these properties makes this probe a prospective versatile tool for investigating DNA interactions in biophysical and bioanalytical studies.

## Supplementary Material

Supplementary information
