# Supplementary material for: Solvatochromic fluorene-linked nucleoside and DNA as color-changing fluorescent probes for sensing interactions
Source: Chem Sci. 2016 Jun 21;7(9):5775–85. doi: 10.1039/c6sc02548j (PMC6021979; doi:10.1039/c6sc02548j)
Supplement: Supplementary file 1 [file SC-007-C6SC02548J-s001.pdf]

Electronic Supplementary Material (ESI) for Chemical Science.
This journal is © The Royal Society of Chemistry 2016

Electronic Supporting Information
for

Solvatochromic Fluorene-Linked Nucleoside and DNA as Color-Changing Fluorescent Probes for Sensing Interactions

Dmytro Dziuba,[a] Petr Pospíšil,[b] Ján Matyašovský,[a] Jiří Brynda,[a] Dana Nachtigallová,[a] Lubomír Rulíšek,[a] Radek Pohl,[a] Martin Hof,[b] Michal Hocek[ac*]

[a] Institute of Organic Chemistry and Biochemistry, Academy of Sciences of the Czech Republic, Gilead & IOCB Research Center, Flemingovo nam. 2, CZ-16610 Prague 6, Czech Republic
[b] J. Heyrovský Institute of Physical Chemistry, Academy of Sciences of the Czech Republic, v.v.i., Dolejskova 3, CZ-182 23 Prague, Czech Republic
[c] Department of Organic Chemistry, Faculty of Science, Charles University in Prague, Hlavova 8, CZ-12843 Prague 2, Czech Republic
** to whom the correspondence should be addressed: hocek@uochb.cas.cz*

Contents

A. Additional figures 3
B. List of abbreviations 6
C. Experimental - Chemical synthesis 7
Materials and methods 7
N-Boc-*N*-methyl-9,9-dimethyl-2-aminofluorene (3) 7
2-(*N*-acetyl, *N*-methyl-amino)-9,9-dimethyl-fluorene (5) 8
2-(*N*-acetyl, *N*-methyl-amino)-7-acetyl-9,9-dimethyl-fluorene (6) 8
2-Methylamino-7-acetyl-9,9-dimethyl-fluorene (7) 9
2-(*N*-propargyl, *N*-methyl-amino)-7-acetyl-9,9-dimethyl-fluorene (8) 9
5-{3-[(7-acetyl-9,9-dimethyl-fluoren-2-yl)(methyl)amino]propyn-1-yl}-2'-deoxycytidine (dC^{FL}, 9) 10
5-{3-[(7-acetyl-9,9-dimethyl-fluoren-2-yl)(methyl)amino]propyn-1-yl}-2'-deoxycytidine-5'-O-triphosphate ($dC^{FL}TP$, 10) 11
D. Experimental - Enzymatic synthesis and characterization of modified DNA 12
Materials and methods 12
Analytical primer extension 12
Semi-preparative primer extension with magnetic separation 12
Polymerase chain reaction (PCR) 13

Semi-preparative preparation of DNA^{FL} 13
Circular dichroism 13
EMSA with p53CD_GST 14
E. Absorption and steady-state fluorescence measurements 15
Materials and methods 15
Determination of fluorescence quantum yields 15
Preparation of DOTAP liposomes 15
Binding of DNA^{FL} to p53CD_GST 15
Binding of DNA^{FL} to DOTAP 16
F. Computational Details 17
General methods 17
Molecular modeling 17
G. TDFS measurements 18
Instrumentation 18
Method 18
Sample preparation and measurements 19
H. Image analysis 21
Image acquisition and processing 21
Compound 8 in 1,4-dioxane/water mixtures 21
I. References 23
J. Copies of NMR spectra 24

A. Additional figures

(a) (b)

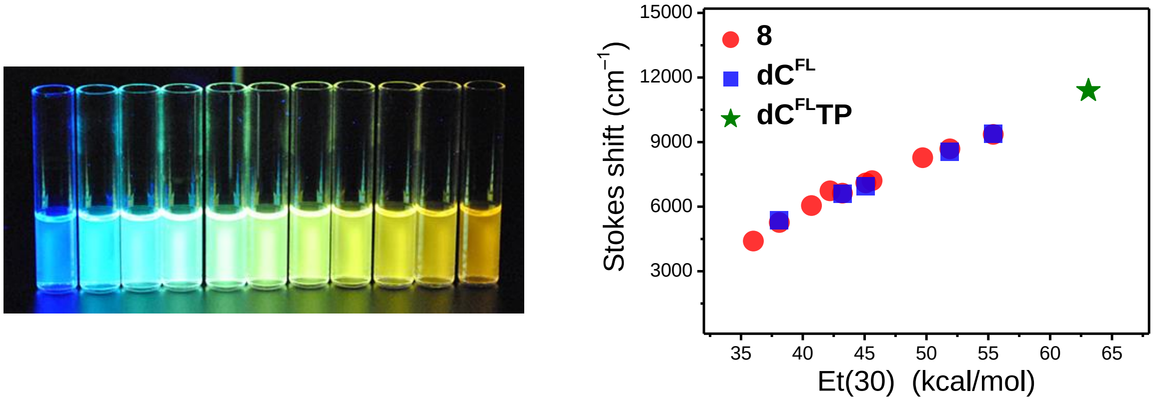

Figure S1. (**a**) Photograph of solutions of the solvatochromic fluorene (**8**) in 1,4-dioxane (left), water (right) and dioxane-water mixtures of variable composition (in the middle); the photograph was taken under a UV-lamp; see also Figures S6–S8 below. (**b**) Correlation of the Stokes shift of compounds **8**, **dC^{FL}** and **dC^{FL}TP** with the empirical solvent polarity scale Et(30)

(a) (b)

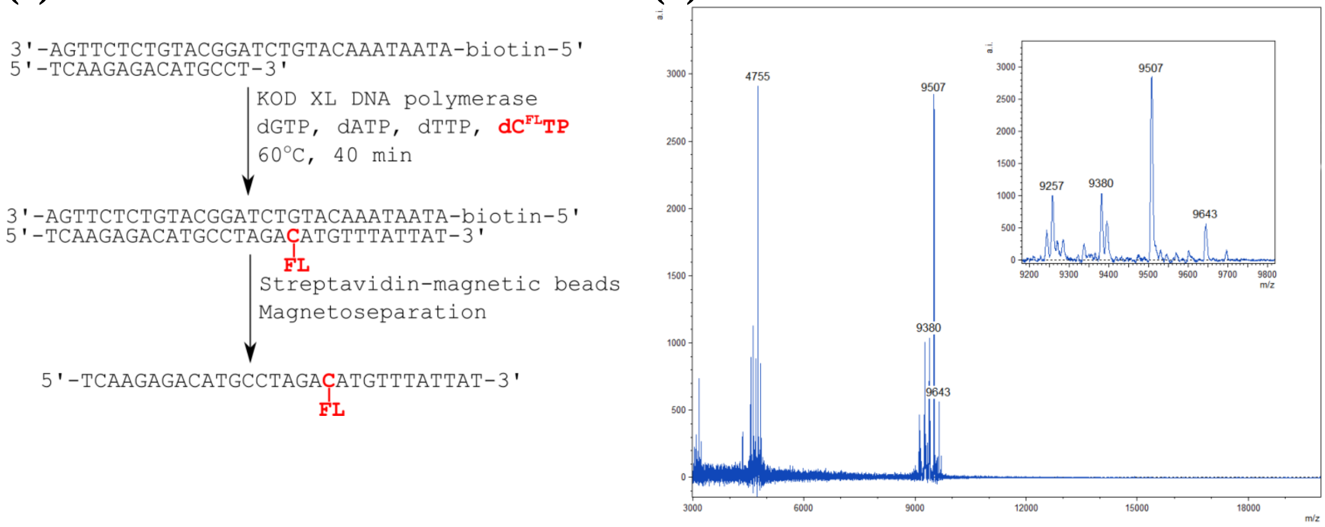

Figure S2. PEX followed by magneto-separation and MALDI mass-spectrometry proves the incorporation of **dC^{FL}TP** into DNA by KOD XL DNA polymerase. (**a**) Scheme of the preparation of ssDNA by primer extension with 5'-biotinylated template followed by magneto-separation with streptavidin magnetic beads; fluorene-modified nucleoside shown in red. (**b**) MALDI spectrum of the obtained fluorene-labelled ssDNA; MS for [M+H] *m/z* calculated = 9506 Da, found = 9507 Da; the peak at *m/z* = 9643 corresponds to the residual signal of the biotinylated template; peaks at [M – (n×125)] can be assigned to dethymination products formed during ionization.

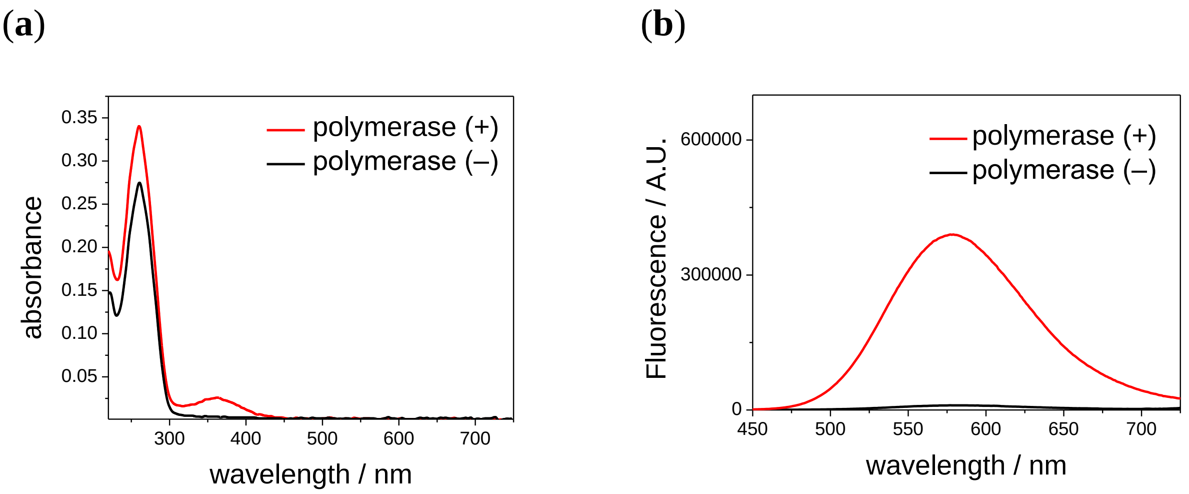

Figure S3. (**a**) UV-vis absorption spectra and (**b**) fluorescence spectra (λ_{ex}=370 nm) of DNA obtained after incubation of PEX reaction mixtures containing **dC^{FL}TP**, dATP, dTTP, dGTP, primer 5'-GAATTCGATATCAAGAGACATGCCT-3' and template 5'-TACCTTATCCATAATAGACATGTCT AGGCATGTCTCTTGATATCGAATTC-3' either with (red line) or without (black line) KOD XL DNA polymerase; the reaction mixtures were incubated at 60 °C for 30 min, then the reactions were stopped by cooling on ice; DNA from solutions was isolated using QIAquick Nucleotide Removal Kit (QIAGEN). The difference between two samples indicates that **dC^{FL}TP** is accepted as a substrate by DNA polymerase and does not bind unspecifically to DNA.

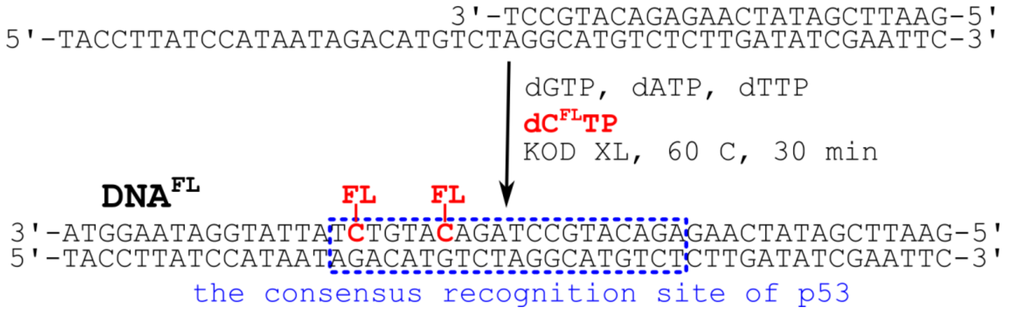

Scheme S1. Preparation of **DNA^{FL}** by PEX with KOD XL DNA polymerase in the presence of **dC^{FL}TP** and three remaining dNTPs; the positions of fluorene-modified deoxycytidines are shown in red; blue frame shows the consensus recognition site of the human protein p53 (ref. S1). Natural control DNA was prepared from the same template and primer by PEX in the presence of the four natural dNTPs.

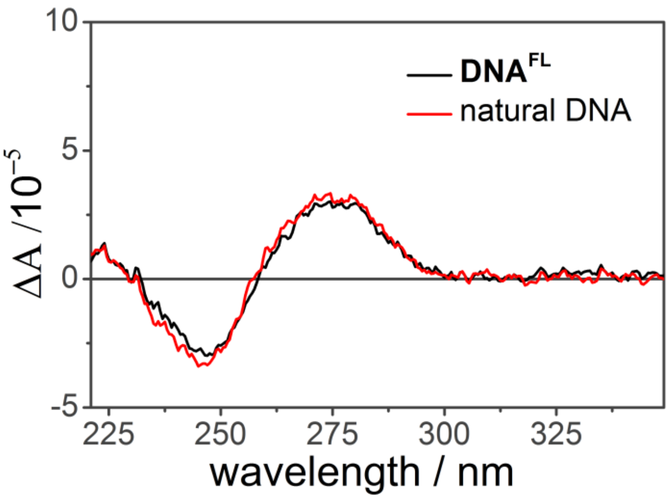

Figure S4. CD spectra of double-stranded **DNA^FL** in comparison with natural non-modified control DNA; conditions: C_{DNA} = 1 μM, 10 mM sodium phosphate buffer pH 7.4, 200 mM NaCl, t = 25 °C.

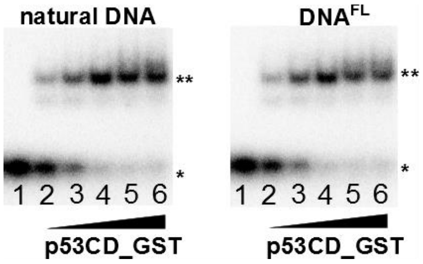

Figure S5. Binding of **p53CD_GST** to non-modified natural DNA (left) and **DNA^FL** (right) observed by EMSA; free (*) and p53-bound (**) bands are indicated. Concentration of DNA was of 0.18 μM; concentration of **p53CD_GST** in lanes 1–6 was 0, 0.21, 0.42, 0.63, 0.84 and 1.05 μM, respectively.

B. List of abbreviations

CD – Circular dichroism

dNTP – Deoxynucleotide triphosphate

DOTAP – 1,2-Dioleoyl-3-trimethylammonium-propane

DTT – Dithiothreitol

EDTA – Ethylenediaminetetraacetic acid

EMSA – Electrophoretic mobility shift assay

fwhm – Full width at half maximum

HPLC – High performance liquid chromatography

IRF – Instrument response function

MALDI – Matrix assisted laser desorption ionization

PAGE – Polyacrylamide gel electrophoresis

PBS – Phosphate buffered saline

PCR – Polymerase chain reaction

PEX – Primer extension

SCM – Spectral center of mass

SUV – Small unilamellar vesicles

TBE – Tris borate – EDTA buffer

TCSPC – Time-correlated single photon counting

TDFS – Time dependent fluorescence shift

TEAB – Triethylammonium bicarbonate buffer

TLC – Thin layer chromatography

TRES – Time resolved emission spectra

C. Experimental - Chemical synthesis

Materials and methods

Reagents and solvents were purchased from Sigma–Aldrich and AlfaAesar. 5-Iodo-2'-deoxycytidine (**dC^{I}**) was purchased from Berry&Associates. *N*-Boc-2-aminofluorene (**2**) was prepared from 2-aminofluorene (**1**) as described in the literature.[S2] Column chromatography was performed by using silica gel (40–63 μm). Purification of **dC^{FL}TP** was performed using HPLC (Waters modular HPLC system) on a column packed with 10 μm C18 reversed phase (Phenomenex, Luna C18 (2) 100 Å). NMR spectra were measured on a Bruker AVANCE 500 (^{1}H at 500.0 MHz, ^{13}C at 125.7 MHz and ^{31}P at 202.3 MHz) and Bruker AVANCE 600 (^{1}H at 600.1 MHz and ^{13}C at 150.9 MHz) NMR spectrometers in $CDCl_3$, DMSO-d_6 or D_2O solutions. Chemical shifts (in ppm, δ scale) were referenced to the residual solvent signal in ^{1}H spectra (δ($CHCl_3$) = 7.26 ppm, δ((CHD_2)SO(CD_3)) = 2.5 ppm) or to the solvent signal in ^{13}C spectra (δ($CDCl_3$) = 77.0 ppm, δ((CD_3)$_2$SO) = 39.7 ppm). 1,4-Dioxane was used as an internal standard for D_2O solutions (3.75 ppm for ^{1}H and 69.3 ppm for ^{13}C). ^{31}P NMR spectra were referenced to phosphate buffer signal (2.35 ppm). Coupling constants (*J*) are given in Hz. The complete assignment of ^{1}H and ^{13}C signals was performed by an analysis of the correlated homonuclear H,H-COSY, and heteronuclear H,C-HSQC and H,C-HMBC spectra. High resolution mass spectra were measured on a LTQ Orbitrap XL spectrometer (Thermo Fisher Scientific).

N-Boc-*N*-methyl-9,9-dimethyl-2-aminofluorene (3)

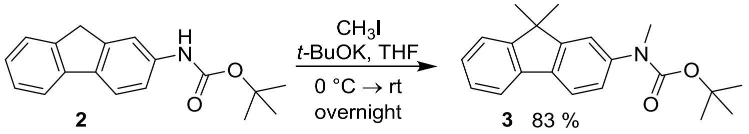

Potassium *tert*-butoxide (3.12 g, 4 equiv) was added by small portions during 15 min to a stirred ice-cooled solution of *N*-Boc-2-aminofluorene **2**[S2] (1.96 g, 6.98 mmol) and methyl iodide (1.95 mL, 4.5 equiv) in THF (50 mL). The water–ice bath was removed and the reaction mixture was stirred overnight at ambient temperature. Then the reaction was quenched with water and extracted with dichloromethane (3×). The combined organic layers were dried over $MgSO_4$ and concentrated on a rotavap. The viscous residue was subjected to column chromatography purification eluting with EtOAc in hexane (0→11% v/v). Appropriate fractions were combined, concentrated on a rotavap and dried in high vacuum until constant mass to give the product as a viscous colorless oil (1.87 g, 83%). ^{1}H NMR (500.0 MHz, $CDCl_3$): 1.46 (bs, 9H, (CH_3)$_3$C); 1.48 (s, 6H, CH_3-9); 3.32 (s, 3H, CH_3N); 7.17 (bdd, 1H, $J_{3,4}$= 8.1, $J_{3,1}$ = 2.0, H-3); 7.29 (bd, 1H, $J_{1,3}$ = 2.0, H-1); 7.30 (td, 1H, $J_{7,6}$ = $J_{7,8}$ = 7.4, $J_{7,5}$ = 1.4, H-7); 7.33 (td, 1H, $J_{6,5}$ = $J_{6,7}$ = 7.4, $J_{6,8}$ = 1.4, H-6); 7.42 (m, 1H, H-8); 7.65 (dd, 1H, $J_{4,3}$ = 8.1, $J_{4,1}$ = 0.4, H-4); 7.68 (m, 1H, H-5). ^{13}C NMR (125.7 MHz, $CDCl_3$): 27.12 (CH_3-9); 28.36 ((**C**H$_3$)$_3$C); 37.58 (CH_3N); 46.85 (C-9); 80.19 (**C**(CH_3)$_3$); 119.79, 119.81 (CH-4,5); 120.34 (CH-1); 122.54 (CH-8); 123.93 (CH-3); 126.97 (CH-6,7); 136.37 (C-4a); 138.69 (C-4b); 143.05 (C-2); 153.69 (C-8a); 153.90 (C-9a); 154.86 (CO). HRMS (EI): calculated for $C_{21}H_{25}NO_2$ $[M]^+$: 323.1885; found: 323.1886.

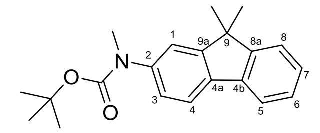

2-(*N*-acetyl, *N*-methyl-amino)-9,9-dimethyl-fluorene (5)

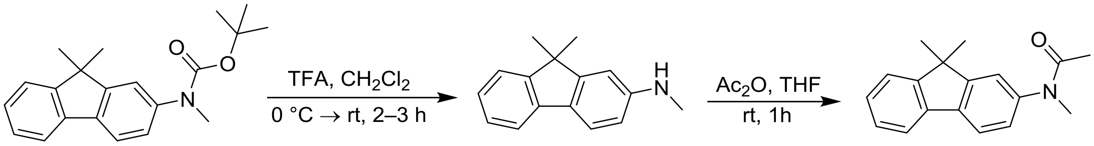

3 4 5 96% over 2 steps

Trifluoroacetic acid (1.6 mL, 10 equiv) was added dropwise to a stirred ice-cooled solution of compound **3** (685 mg, 2.12 mmol) in dichloromethane (18 mL). The solution was stirred for 30 min at 0 °C and then at ambient temperature for 2–3 hours, until complete conversion of the starting material was evidenced by TLC (EtOAc in hexane, 10% v/v). The reaction was quenched with saturated aqueous $NaHCO_3$ (*Caution!* Evolution of CO_2). The organic layer was separated; the aqueous layer was extracted with CH_2Cl_2 (3×). The combined organic layers were dried over $MgSO_4$ and evaporated to dryness on a rotavap to give intermediate **4** as viscous oil which was used in the next steps without further purification. Intermediate **4** was dissolved in THF (15 mL) and then acetic anhydride (0.4 mL, 2 equiv) was added. The reaction mixture was stirred at ambient temperature for 1 hour. TLC (EtOAc in hexane, 30% v/v) showed complete conversion of the starting material. The reaction was quenched with saturated aqueous $NaHCO_3$ and stirred for 1 hour at room temperature. Then the mixture was extracted with CH_2Cl_2 (3×), the combined organic layers were dried over $MgSO_4$ and concentrated on a rotavap; the crude product was purified by column chromatography eluting with EtOAc in hexane (20→60% v/v). The product was obtained as a viscous oil which solidified upon standing; 542 mg (96% over two steps). ^{1}H NMR (500.0 MHz, $CDCl_3$): 1.49 (s, 6H, CH_3-9); 1.92 (s, 3H, CH_3CO); 3.32 (s, 3H, CH_3N); 7.14 (bdd, 1H, $J_{3,4}$= 7.9, $J_{3,1}$ = 1.8, H-3); 7.23 (bd, 1H, $J_{1,3}$ = 1.8, H-1); 7.33 (td, 1H, $J_{6,5}$ = $J_{6,7}$ = 7.4, $J_{6,8}$ = 1.7, H-6); 7.35 (td, 1H, $J_{7,6}$ = $J_{7,8}$ = 7.4, $J_{7,5}$ = 1.6, H-7); 7.44 (m, 1H, H-8); 7.71 (m, 1H, H-5); 7.72 (d, 1H, $J_{4,3}$ = 7.9, H-4). ^{13}C NMR (125.7 MHz, $CDCl_3$): 22.36 (**C**H_3CO); 26.97 (CH_3-9); 37.32 (CH_3N); 46.92 (C-9); 120.07 (CH-5); 120.77 (CH-4); 121.21 (CH-1); 122.59 (CH-8); 125.76 (CH-3); 127.10 (CH-7); 127.61 (CH-6); 137.92 (C-4b); 138.64 (C-4a); 143.48 (C-2); 153.63 (C-8a); 155.22 (C-9a); 170.68 (CO). HRMS (EI): calculated for $C_{18}H_{19}NO$ $[M]^+$: 265.1467; found: 265.1470.

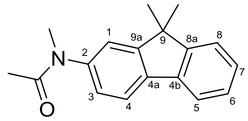

2-(*N*-acetyl, *N*-methyl-amino)-7-acetyl-9,9-dimethyl-fluorene (6)

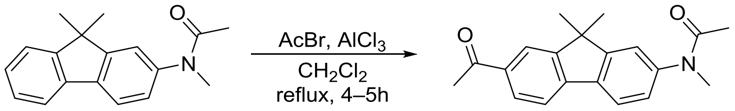

5 6 84%

Acetyl bromide (123 μL, 1.64 mmol) was added to a stirred suspension of $AlCl_3$ (654 mg, 4.92 mmol) in dry dichloromethane (15 mL) and the resulting mixture was stirred for 10 minutes. A solution of fluorene **5** (725 mg, 2.73 mmol) in dichloromethane (6 mL) was then added dropwise. The reaction mixture was refluxed for 4 hours while new portions of acetyl bromide (123 μL, 1.64 mmol) were added after 1 and 2 hours. If the starting material was still detected in the reaction mixture by TLC after 4 hours, a fresh portion of $AlCl_3$ (0.6 – 1.2 mmol) was added and the reaction mixture was refluxed for one more hour. The reaction was cooled down and quenched by slow addition of saturated aqueous $NaHCO_3$. The organic phase was separated; the aqueous phase was extracted with CH_2Cl_2 (3×). The combined organic layers were dried over

$MgSO_4$ and concentrated on a rotavap. The crude product was purified by column chromatography eluting with EtOAc in hexane (30→80% v/v). The title compound was obtained as a white solid (705 mg, 84%). ^{1}H NMR (600.1 MHz, $CDCl_3$): 1.54 (s, 6H, CH_3-9); 1.93 (bs, 3H, CH_3CON); 2.67 (s, 3H, CH_3CO); 3.33 (bs, 3H, CH_3N); 7.20 (dd, 1H, $J_{3,4}$ = 8.0, $J_{3,1}$ = 1.8, H-3); 7.28 (d, 1H, $J_{1,3}$ = 1.8, H-1); 7.79 (d, 1H, $J_{5,6}$ = 7.9, H-5); 7.81 (d, 1H, $J_{4,3}$ = 8.0, H-4); 7.99 (dd, 1H, $J_{6,5}$ = 7.9, $J_{6,8}$ = 1.6, H-6); 8.07 (dd, 1H, $J_{8,6}$ = 1.6, $J_{8,5}$ = 0.5, H-8). ^{13}C NMR (150.9 MHz, $CDCl_3$): 22.48 (**C**H_3CON); 26.79 (**C**H_3CO); 26.87 (CH_3-9); 37.33 (CH_3N); 47.21 (C-9); 119.99 (CH-5); 121.52 (CH-1); 121.93 (CH-4); 122.42 (CH-8); 126.21 (CH-3); 128.38 (CH-6); 136.39 (C-7); 137.32 (C-4a); 142.84 (C-4b); 144.82 (C-2); 153.99 (C-8a); 156.53 (C-9a); 170.50 (CH_3**C**ON); 197.83 (CH_3**C**O). HRMS (EI): calculated for $C_{20}H_{21}NO_2$ $[M]^+$: 307.1572; found: 307.1573.

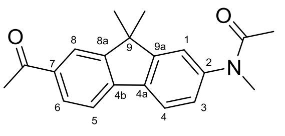

2-Methylamino-7-acetyl-9,9-dimethyl-fluorene (7)

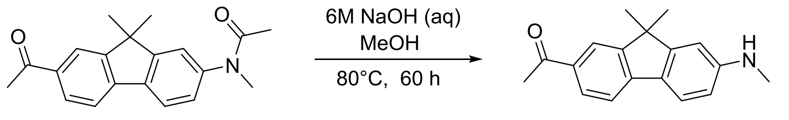

6 **7** 81%

Aqueous NaOH (6M, 8 mL) was added to a stirred solution of compound **6** (2.10 g) in methanol (37 mL) and the resulting mixture was stirred at 80 °C for 2.5 days (ca. 60 hours). Then the reaction was cooled down to room temperature, diluted with water (100 mL) and extracted with CH_2Cl_2 (3×). The combined organic layers were dried over $MgSO_4$, and concentrated on a rotavap. The crude mixture was purified by column chromatography eluting with EtOAc in hexane (5→24% v/v) to give the product as a yellow solid; 1.47 g (81%). ^{1}H NMR (500.0 MHz, DMSO-d_6): 1.41 (s, 6H, CH_3-9); 2.58 (s, 3H, CH_3CO); 2.76 (d, 3H, J = 5.0, CH_3N); 6.08 (bq, 1H, J = 5.0, NH); 6.55 (dd, 1H, $J_{3,4}$= 8.3, $J_{3,1}$ = 2.1, H-3); 6.67 (d, 1H, $J_{1,3}$ = 2.1, H-1); 7.61 (d, 1H, $J_{4,3}$ = 8.3, H-4); 7.65 (dd, 1H, $J_{5,6}$ = 8.0, $J_{5,8}$ = 0.5, H-5); 7.88 (dd, 1H, $J_{6,5}$ = 8.0, $J_{6,8}$ = 1.6, H-6); 7.98 (ddd, 1H, $J_{8,6}$ = 1.6, $J_{8,5}$ = 0.5, H-8). ^{13}C NMR (125.7 MHz, DMSO-d_6): 26.88 (**C**H_3CO); 27.20 (CH_3-9); 29.97 (CH_3N); 46.30 (C-9); 105.23 (CH-1); 111.47 (CH-3); 117.86 (CH-5); 122.10 (CH-8); 122.32 (CH-4); 125.26 (C-4a); 128.51 (CH-6); 133.62 (C-7); 145.07 (C-4b); 151.40 (C-2); 152.37 (C-8a); 156.85 (C-9a); 197.36 (**C**O). HRMS (EI): calculated for $C_{18}H_{19}NO$ $[M]^+$: 265.1467; found: 265.1466.

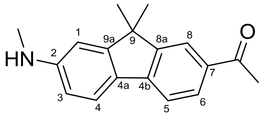

2-(*N*-propargyl, *N*-methyl-amino)-7-acetyl-9,9-dimethyl-fluorene (8)

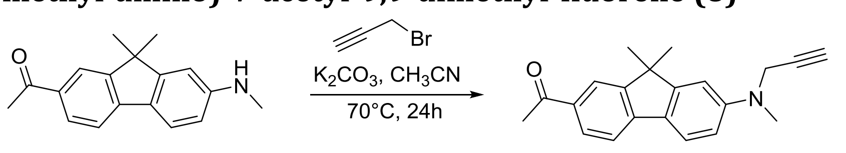

7 **8** 82%

Propargyl bromide (80% w/w solution in toluene, 1.33 mmol, 0.15 mL) was added to a stirred suspension of fluorene **7** (253 mg, 0.95 mmol) and K_2CO_3 (200 mg, 1.44 mmol) in dry acetonitrile (5 mL). The resulting mixture was stirred at 70 °C for 24 hours. Then the reaction was quenched with water and extracted with dichloromethane (3×). The combined organic layers were dried over $MgSO_4$ and concentrated on a rotavap. The residue was purified by column chromatography eluting with EtOAc in hexane (5→20% v/v) to give

the product as a yellow solid (239 mg, 82%). ^{1}H NMR (500.0 MHz, $CDCl_3$): 1.50 (s, 6H, CH_3-9); 2.24 (t, 1H, ^{4}J = 2.4, HC≡C); 2.64 (s, 3H, CH_3CO); 3.09 (s, 3H, CH_3N); 4.14 (d, 2H, ^{4}J = 2.4, CH_2N); 6.88 (dd, 1H, $J_{3,4}$ = 8.4, $J_{3,1}$ = 2.4, H-3); 6.91 (d, 1H, $J_{1,3}$ = 2.4, H-1); 7.63 (dd, 1H, $J_{5,6}$ = 7.9, $J_{5,8}$ = 0.6, H-5); 7.66 (d, 1H, $J_{4,3}$ = 8.4, H-4); 7.92 (dd, 1H, $J_{6,5}$ = 7.9, $J_{6,8}$ = 1.6, H-6); 8.00 (dd, 1H, $J_{8,6}$ = 1.6, $J_{8,5}$ = 0.6, H-8). ^{13}C NMR (125.7 MHz, $CDCl_3$): 26.67 (**C**H_3CO); 27.16 (CH_3-9); 38.97 (CH_3N); 42.70 (CH_2N); 46.88 (C-9); 72.42 (H**C**≡C-); 78.83 (-**C**≡CH); 108.06 (CH-1); 113.17 (CH-3); 118.34 (CH-5); 121.84 (CH-4); 122.04 (CH-8); 128.47 (C-4a, CH-6); 134.50 (C-7); 144.67 (C-4b); 149.55 (C-2); 153.12 (C-8a); 156.63 (C-9a); 197.87 (CO). HRMS (APCI): calculated for $C_{21}H_{22}NO$ $[M+H]^+$: 304.1696; found: 304.1696.

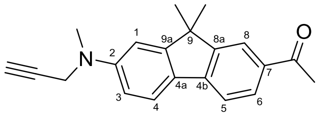

5-{3-[(7-acetyl-9,9-dimethyl-fluoren-2-yl)(methyl)amino]propyn-1-yl}-2'-deoxycytidine (dC^{FL}, 9)

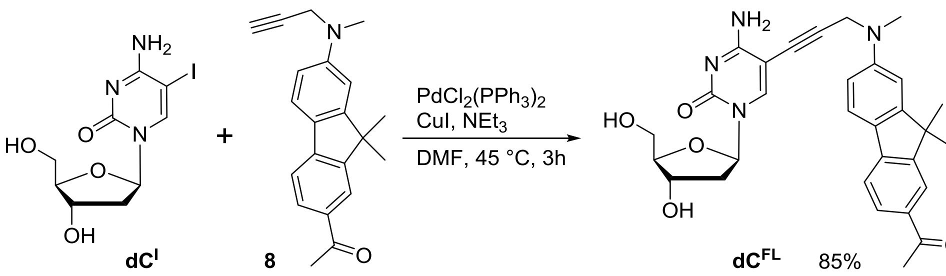

Dry DMF (1.5 ml) was added to a flask containing 5-iodo-2'-deoxycitidine (60 mg, 0.17 mmol), acetylene **8** (62 mg, 0.20 mmol), $PdCl_2(PPh_3)_2$ (6 mg, 5% mol), CuI (2 mg, 5% mol) and the resulting mixture was purge-and-refilled with argon 3 times. Triethylamine (47 µl, 0.34 mmol) was added via syringe and the mixture was stirred at 45 °C until the complete consumption of the starting nucleoside was observed by TLC (ca 3 hours). Then the reaction mixture was filtered through a pad of Celite and evaporated on a rotavap. The residue was redissolved in MeOH, coevaporated with silica gel and purified by silica gel column chromatography eluted with methanol in dichloromethane (0→7% v/v) to afford the desired nucleoside as a yellow solid (76 mg, 85%). ^{1}H NMR (600.1 MHz, DMSO-d_6): 1.45 (s, 6H, CH_3-9"); 1.95 (ddd, 1H, J_{gem} = 13.1, $J_{2'b,1'}$ = 7.1, $J_{2'b,3'}$ = 6.1, H-2′b); 2.11 (ddd, 1H, J_{gem} = 13.1, $J_{2'a,1'}$ = 6.0, $J_{2'a,3'}$ = 3.6, H-2′b); 2.59 (s, 3H, CH_3CO); 3.05 (s, 3H, CH_3N); 3.52 (ddd, 1H, J_{gem} = 11.9, $J_{5'b,OH}$ = 5.2, $J_{5'b,4'}$ = 3.6, H-5′b); 3.58 (ddd, 1H, J_{gem} = 11.9, $J_{5'a,OH}$ = 5.2, $J_{5'a,4'}$ = 3.6, H-5′a); 3.76 (q, 1H, $J_{4',3'}$ = $J_{4',3'}$ = 3.6, H-4′); 4.17 (ddt, 1H, $J_{3',2'}$ = 6.1, 3.6, $J_{3',OH}$ = 4.3, $J_{3',4'}$ = 3.6, H-3′); 4.46 (s, 2H, CH_2N); 5.01 (t, 1H, $J_{OH,5'}$ = 5.2, OH-5′); 5.18 (d, 1H, $J_{OH,3'}$ = 4.3, OH-3′); 6.07 (dd, 1H, $J_{1',2'}$ = 7.1, 6.0, H-1′); 6.68 (bs, 1H, NH_a**H**$_b$); 6.91 (dd, 1H, $J_{3'',4''}$ = 8.4, $J_{3'',1''}$ = 2.4, H-3″); 7.10 (d, 1H, $J_{1'',3''}$ = 2.4, H-1″); 7.73 (d, 1H, $J_{4'',3''}$ = 8.4, H-4″); 7.74 (d, 1H, $J_{5'',6''}$ = 7.9, H-5″); 7.75 (bs, 1H, N**H**$_a$$H_b$); 7.91 (dd, 1H, $J_{6'',5''}$ = 7.9, $J_{6'',8''}$ = 1.6, H-6″); 8.01 (dd, 1H, $J_{8'',6''}$ = 1.6, $J_{8'',5''}$ = 0.5, H-8″); 8.09 (s, 1H, H-6). ^{13}C NMR (150.9 MHz, DMSO-d_6): 26.93 (**C**H_3CO); 27.07 (CH_3-9"); 38.83 (CH_3N); 40.90 (CH_2-2′); 42.82 (CH_2N); 46.68 (C-9"); 61.18 (CH_2-5′); 70.27 (CH-3′); 75.58 (cyt-**C**≡C-); 85.56 (CH-1′); 87.62 (CH-4′); 89.57 (C-5); 91.76 (**C**≡C-cyt); 107.90 (CH-1″); 112.91 (CH-3″); 118.56 (CH-5″); 122.14 (CH-4″); 122.19 (CH-8″); 126.89 (C-4″a); 128.44 (CH-6″); 134.24 (C-7″); 144.39 (CH-6); 144.46 (C-4″b); 150.02 (C-2″); 152.96 (C-8″a); 153.58 (C-2); 156.57 (C-9″a); 164.57 (C-4); 197.52 (CO).HRMS (ESI): calculated for $C_{30}H_{32}O_5N_4Na$ $[M+Na]^+$: 551.2265; found: 551.2264.

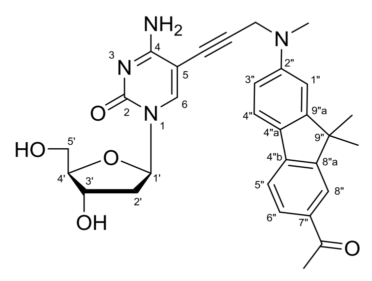

5-{3-[(7-acetyl-9,9-dimethyl-fluoren-2-yl)(methyl)amino]propyn-1-yl}-2'-deoxycytidine-5'-O-triphosphate ($dC^{FL}TP$, 10)

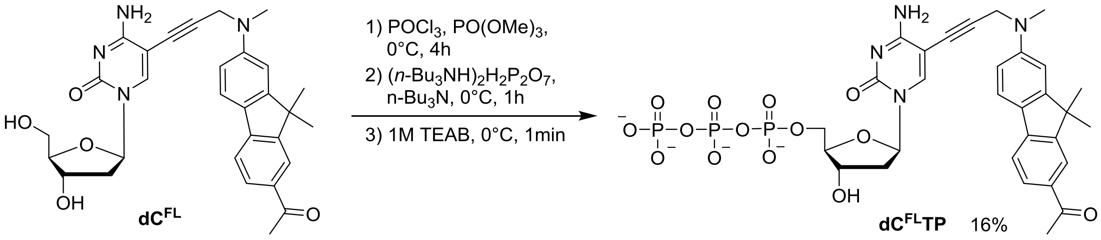

Dry trimethyl phosphate (0.8 mL) was added to an argon-purged flask containing nucleoside **dC^{FL}** (36 mg, 0.068 mmol). The resulting solution was cooled down to 0 ºC and a solution of $POCl_3$ (10 µL, 0.108 mmol) in dry trimethyl phosphate (0.8 mL) was added dropwise. After 4 hours stirring at 0 ºC, a solution of $(n\text{-}Bu_3NH)_2H_2P_2O_7$ (168 mg, 0.306 mmol) and n-Bu_3N (73 µL, 0.306 mmol) in dry DMF (0.8 mL) was added dropwise. The reaction mixture was stirred for another 60 min at 0 ºC and then quenched by the addition of cold 1M TEAB (2 mL). The mixture was concentrated on a rotavap; the residue was co-evaporated with distilled water three times. The crude product was dissolved in water (ca. 3 mL) and unreacted nucleoside was separated by filtration. The aqueous solution was purified by semi-preparative HPLC using a linear gradient of methanol (5→100%) in 0.1 M TEAB buffer. The appropriate fractions were combined and evaporated on a rotavap. The viscous yellow oil was co-evaporated with distilled water three times. The product was converted to sodium salt on an ion-exchange column (Dowex 50WX8 in Na^+ cycle) and freeze-dried. The title compound was obtained as yellow solid (9.3 mg, 16%). ^{1}H NMR (500.0 MHz, D_2O, pD = 7.1 (phosphate buffer), ref(dioxane) = 3.75 ppm): 1.26, 1.27 (2 × s, 2 × 3H, CH_3-9"); 1.88 (dt, 1H, J_{gem} = 13.7, $J_{2'b,1'}$ = $J_{2'b,3'}$ = 6.6, H-2′b); 2.15 (ddd, 1H, J_{gem} = 13.7, $J_{2'a,1'}$ = 6.6, $J_{2'a,3'}$ = 4.3, H-2′b); 2.48 (s, 3H, CH_3CO); 3.00 (s, 3H, CH_3N); 3.99 – 4.14 (m, 3H, H-4′,5′); 4.35 – 4.41 (m, 3H, H-3′, CH_2N); 5.82 (t, 1H, $J_{1',2'}$ = 6.6, H-1′); 6.59 (dd, 1H, $J_{3'',4''}$ = 8.4, $J_{3'',1''}$ = 2.4, H-3″); 6.81 (d, 1H, $J_{5'',6''}$ = 7.9, H-5″); 6.96 (d, 1H, $J_{4'',3''}$ = 8.4, H-4″); 7.07 (d, 1H, $J_{1'',3''}$ = 2.4, H-1″); 7.38 (d, 1H, $J_{6'',5''}$ = 7.9, H-6″); 7.68 (s, 1H, H-6); 7.82 (d, 1H, $J_{8'',6''}$ = 1.6, H-8″). ^{13}C NMR (125.7 MHz, D_2O, pD = 7.1 (phosphate buffer), ref(dioxane) = 69.30 ppm): 28.78 (**C**H_3CO); 28.84, 28.89 (CH_3-9"); 41.52 (CH_2-2′); 41.76 (CH_3N); 46.06 (CH_2N); 49.33 (C-9"); 67.85 (d, $J_{C,P}$ = 5.1, CH_2-5′); 72.86 (CH-3′); 77.16 (cyt-**C**≡C-); 87.93 (d, $J_{C,P}$ = 8.3, CH-4′); 88.92 (CH-1′); 94.74 (C-5); 95.02 (**C**≡C-cyt); 111.92 (CH-1″); 116.85 (CH-3″); 121.31 (CH-5″); 124.94 (CH-4″); 125.03 (CH-8″); 130.98 (C-4″a); 131.33 (CH-6″); 136.18 (C-7″); 146.80 (CH-6); 147.58 (C-4″b); 152.69 (C-2″); 155.94 (C-8″a); 158.01 (C-2); 159.68 (C-9″a); 167.20 (C-4); 205.34 (CO). ^{31}P{^{1}H} NMR (202.3 MHz, D_2O, pD = 7.1 (phosphate buffer), ref(phosphate buffer) = 2.35 ppm): – 21.14 (t, J = 19.3, P_β); – 10. 29 (d, J = 19.3, P_α); – 6.78 (d, J = 19.3, P_γ). HRMS (ESI): calculated for $C_{30}H_{32}O_{14}N_4Na_2P_3$ $[M+2Na+H]^-$: 811.0929; found: 811.0918.

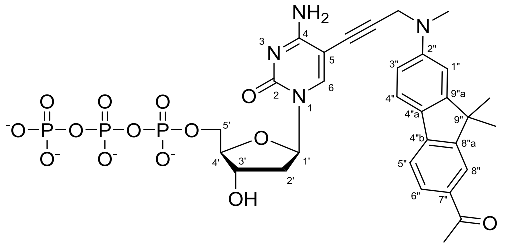

D. Experimental - Enzymatic synthesis and characterization of modified DNA

Materials and methods

Oligonucleotides were purchased from Generi Biotech (Czech Republic). Double-stranded 100bp DNA ladder for PCR was purchased from New England Biolabs. Bst DNA polymerase (Large Fragment) and corresponding reaction buffer, as well as natural nucleoside triphosphates (dATP, dCTP, dGTP, dTTP) were purchased from New England Biolabs. KOD XL DNA polymerase and corresponding reaction buffer were obtained from Merck Millipore. Streptavidin magnetic beads were obtained from Roche. All solutions for biochemical reactions were prepared using Milli-Q water. Primer for analytical primer extension and EMSA experiments was labeled using T4 polynucleotide kinase (New England Biolabs) and [γ^{32}P]-ATP (Institute of Isotopes Co., Ltd.; Hungary) according to standard techniques. Radioactive gels were visualized by phosphorimaging using Storage Phosphor Screens and Typhoon FLA 9500 imager (GE Healthcare). Concentration of DNA solutions were calculated using A_{260} values measured on a Nanodrop and values obtained with OligoCalc.[S3] Mass spectra of oligonucleotides were measured by MALDI-TOF, on UltrafleXtreme MALDI-TOF/TOF mass spectrometer (Bruker Daltonics, Germany), with 1 kHz smartbeam II laser. p53 core domains (p53CD, residues 94–312) with a GST tag (**p53CD_GST**) was expressed from plasmids pGEX-2TK (Roche Diagnostics GmbH) in *E.coli* and purified as described elsewhere.[S4] Final step of purification was dialysis to storage buffer (25mM Hepes pH 7.6, 200mM KCl, 10% glycerol, 1mM DTT, 1mM benzamidine).

Analytical primer extension

The reaction mixture (20 μL) contained primer 5'-[^{32}P]-GAATTCGATATCAAGAGACATG CCT-3' (3 μM, 1 μL), unlabeled primer (100 μM, 0.77 μL), template 5'-TACCTTATCCATAATAGAC ATGTCTAGGCATGTCTCTTGATATCGAATTC-3' (100 μM, 0.8 μL), KOD XL DNA polymerase (0.23 U), either natural or modified dNTPs (4 mM each, 0.4 μL) and reaction buffer (10×, 2 μL) supplied by the manufacturer with the enzyme. The reaction mixture was incubated at 60 °C for 30 min. The reaction was stopped by the addition of PAGE stop solution (40 μL; 80% [v/v] formamide, 20 mM EDTA, 0.025% [w/v] bromophenol blue, 0.025% [w/v] xylene cyanol) and heated at 95 °C for 5 min. Aliquots (3 μL) were subjected to vertical electrophoresis in 12.5% denaturing polyacrylamide gel containing 1× TBE buffer (pH 8.0) and 7 M urea at 50 mA for 40 min. The gel was dried in vacuo (80 °C, 70 min) and visualized by phosphor imaging autoradiography.

Semi-preparative primer extension with magnetic separation

The reaction mixture (100 μL) containing KOD XL DNA polymerase (2.5 U/μL, 0.35 μL), 10× concentrate of the KOD reaction buffer provided by the manufacturer of the enzyme (10 μL), biotinylated template 5'-[biotin]-ATAATAAACATGTCTAGGCATGTCTCTTGA-3' (100 μM, 4 μL), primer 5'-TCAAGAGACATGCCT-3' (100 μM, 4 μL), dNTPs (dGTP, dTTP, dATP, **dC$^{\mathbf{FL}}$TP**; 4 mM each, 5 μL) was incubated at 60 ºC for 40 minutes. The reaction was stoped by cooling to 4 ºC. Streptavidin magnetic beads (100 μl) were washed with binding buffer (3 × 300 μl, 10mM Tris, 1mM EDTA, 100mM NaCl, pH 7.5). Then PEX solution (100 μL) and binding buffer (200 μl) were added to the magnetic beads. The suspension was incubated in a thermal mixer for 35 min at 15 °C and 900 rpm. Then the magnetic beads were separated and washed with ice-cold wash buffer (3 × 300 μl, 10mM Tris, 1mM EDTA, 500mM NaCl, pH 7.5) and ice-cold water (4 × 300 μl). Then water (30 μl) was added and the sample was denatured for 2 min at 41 °C

and 500 rpm. The beads were separated and the solution containing the fluorene-labeled ssDNA was transferred into a clean vial and analysed by MALDI-TOF mass spectrometry.

Polymerase chain reaction (PCR)

The reaction mixture (10 μL) contained KOD XL DNA polymerase (2.5 U/μL, 0.25 μL), 10× concentrate of the KOD reaction buffer supplied by the manufacturer of the enzyme (1 μL), primers 5'-CAAGGACA AAATACCTGTATTCCTT-3' and 5'-GACATCATGAGAGACATCGC-3' (10 μM; 1μL of each), template 5'-GACATCATGAGAGACATCGCCTCTGGGCTAATAGGACTACTTCTAATCTGTAAGA GCAGATCCCTGGACAGGCAAGGAATACAGGTATTTTGTCCTTG-3' (1 μM, 0.25 μL). The amounts of dNTPs depend on whether only natural dNTPs or **dC^{FL}TP** with the three remaining natural dNTPs were used. The following amounts were used: positive control – four natural dNTPs (1 mM of each, 0.6 μL); negative control – three natural dNTPs (4 mM of each dGTP, dTTP, and dATP, 0.6 μL); modified dNTP – **dC^{FL}TP** with three remaining natural dNTPs (4 mM of each **dC^{FL}TP**, dGTP, dTTP, and dATP, 0.6 μL). After the initial denaturation for 3 min at 94 °C, 35 PCR cycles were run under the following conditions: denaturation for 1 min at 94 °C, annealing for 1 min at 51 °C, extension for 2 min at 72 °C. These PCR process was terminated with a final extension step for 5 min at 72 °C. The reaction was stopped by cooling to 4 °C. The PCR products were analyzed by agarose gel electrophoresis in 2% agarose gel stained with GelRed™ (Biotium). Samples for electrophoresis were prepared by mixing 1 μL of 6× DNA Loading Dye (Thermo Scientific) and 5 μL of the reaction mixture. The gel was run for 60 min at 100 V and imaged using a Syngene G:BOX F3 gel documentation system.

Semi-preparative preparation of DNA^{FL}

The reaction mixture (250 μL) contained KOD XL DNA polymerase (2.5 U/μL, 1 μL), 10× concentrate of the KOD reaction buffer provided by the manufacturer of enzyme (25 μL), dNTPs (dTTP, dGTP, dATP, and **dC^{FL}TP**, 4 mM each, 5 μL), primer 5'-GAATTCGATATCAAGAGACATGCCT-3' (100 μM, 10 μL), template 5'-TACCTTATCCATAATAGACATGTCTAGGCATGTCTCTTGATATCGAATTC-3' (100 μM, 10 μL). The reaction mixture was incubated for 30 min at 60 °C in a thermal mixer. The reaction was stopped by cooling to 4 °C. The fluorene-labeled dsDNA was purified using spin columns (QIAquick Nucleotide Removal Kit, QIAGEN); reaction mixture was divided to two columns; the product was eluted from each column by adding 100 μL of water. Concentration of the resulting solutions of **DNA^{FL}** was determined on a NanoDrop.
Natural non-modified DNA for control experiments was prepared following the same protocol with dCTP being used instead of **dC^{FL}TP**.

Circular dichroism

Circular dichroism (CD) experiments were carried out on a Jasco 815 spectropolarimeter (Tokyo, Japan). **DNA^{FL}** and control non-modified DNA (Scheme S1) were measured as 1μM solutions in buffer (10 mM phosphate buffer pH 7.4, 200 mM NaCl). The spectra were collected from 200 nm to 350 nm as averages over 2 scans at room temperature using a 0.1 cm path length. A 0.05 nm step resolution, 5 nm/min speed, 32 sec response time and 1 nm bandwidth were used. Following baseline correction, the spectra were expressed as differential absorption (Figure S4).

EMSA with p53CD_GST

Preparation of [^{32}P]-dsDNA. The reaction mixture (100 μL) contained KOD XL DNA polymerase (2.5 U/μL, 0.4 μL), 10x concentrate of reaction buffer provided by the manufacturer of enzyme (10 μL), dTTP, dGTP, dATP, and either dCTP for non-modified control DNA or **dC^{FL}TP** for **DNA^{FL}** (4 mM each, 2 μL), radioactive primer 5'-[^{32}P]-GAATTCGATATCAAGAGACATGCCT-3' (3 μM, 5 μL), cold primer (100 μM, 3.8 μL), template 5'-TACCTTATCCATAATAGACATGTCTAGGCATGTCTCTTGATATCGAATTC-3' (100 μM, 4 μL). The reaction mixture was incubated for 30 min at 60 °C in a thermal mixer and then stopped by cooling to 4 °C. The dsDNA was purified using QIAquick Nucleotide Removal Kit (QIAGEN), one column /100 μL reaction mixture. The labeled dsDNA was eluted from columns with water (100 μL); 7.5 μL of this concentrated dsDNA solution was diluted with water to 100 μL and the resulting solutions were used in binding studies.

*Binding with **p53CD_GST***. Binding mixture (total volume 20 μL) contained [^{32}P]-dsDNA solution prepared as described above (10 μL), KCl (500mM, 2 μl), DTT (2mM, 2 μl), VP buffer (50mM Tris, 0.1% Triton-X100, pH 7.6, 2 μl) and **p53CD_GST** stock solution (700 ng/μl in 25mM Hepes pH 7.6, 200mM KCl, 10% glycerol, 1mM DTT, 1mM benzamidine; either 0, 0.3, 0.6, 0.9, 1.2 or 1.5 μL) and p53 storage buffer (25mM Hepes pH 7.6, 200mM KCl, 10% glycerol, 1mM DTT, 1mM benzamidine; either 1.5, 1.2, 0.9, 0.6, 0.3, 0 μL). The binding mixtures were incubated on ice for 30–60 min, then mixed with 80% v/v aqueous glycerol (2 μL). Aliquots (3 μL) were analyzed by 5% native vertical PAGE containing 0.5× TBE buffer pH 8.0 (80 V, 1 h at 4 °C). The gels were visualized by phosphor imaging autoradiography.

E. Absorption and steady-state fluorescence measurements

Materials and methods

Chemicals and spectroscopy grade solvents were purchased from Sigma-Aldrich, Alfa Aesar, Acros Organics and used as supplied. 1,2-Dioleoyl-3-methylammonium-propane (DOTAP) were purchased from Avanti Polar Lipids, Inc. (Alabaster, USA). UV-visible spectra were measured on a Cary 100 UV-Vis spectrometer (Agilent Technologies). Fluorescence spectra were measured on a Fluoromax 4 spectrofluorimeter (HORIBA Scientific).

Determination of fluorescence quantum yields

Relative determination of the fluorescence quantum yields[S5,S6] (Φ_f) was performed using quinine sulfate in 0.5 M H_2SO_4 (Φ_f = 0.55 at 25 °C) as a standard.[S7] Measurements were performed in semi-micro quartz fluorescence cuvettes (Hellma Analytics) on a Fluoromax 4 spectrofluorimeter equiped with a thermostated cuvette holder at 25 °C. The solvents used were either of spectroscopy or HPLC grade. The excitation wavelength was 365 nm and the recorded spectral range was 370 – 700 nm for compounds **8** and **dC^{FL}**. The excitation wavelength was 370 nm and the the recorded spectral range was 375 – 735 nm for **dC^{FL}TP**. The absorbance of sample solutions at the excitation wavelength were kept below 0.08 to avoid inner filter effects. The quantum yields were calculated using the following equation[S6]

$$\Phi_{f,x} = \Phi_{f,st} \times \frac{F_x}{F_{st}} \times \frac{1 - 10^{-Abs_{st}}}{1 - 10^{-Abs_x}} \times \frac{n_x^2}{n_{st}^2}$$

Here Φ_f is the quantum yield, *F* is the integrated fluorescence intensity, *Abs* is the absorbance of solution at the excitation wavelength, *n* is the refractive index of solvent; the subscripts *x* and *st* stand for the sample and standard, respectively. Measurements were triplicated; the uncertainty of measured values of quantum yields was ± 0.03.

Preparation of DOTAP liposomes

Appropriate volume of DOTAP stock solution (10 mM in $CHCl_3$) was transferred into a glass vial and then the solvent was evaporated using a stream of nitrogen. The vial was placed into a vacuum desiccator for 20–30 min in order to remove the traces of chloroform. The dried lipid was vortexed (2500 rpm, 5 min) with appropriate volume of PBS (10 mM sodium phosphate buffer pH 7.4, 200 mM NaCl) to reach 1 mM DOTAP concentration. Then sample was sonicated for 10 min using a tip sonicator (Sonopuls HD 2070, Bandelin electronic GmbH, Germany) at 10% power, while cooled with a water bath at room temperature to give small unilamellar vesicles (SUVs). SUVs were centrifuged at 16110g (Eppendorf centrifuge 5415 D) for 20 min to separate the contaminating metallic particles from the sonicator tip. The supernatant was collected and used without any additional treatment.

Binding of DNA^{FL} to p53CD_GST

DNA^{FL} – **p53CD_GST** binding solution contained **DNA^{FL}** (5μM; 3μL), KCl (500mM, 3 μl), DTT (2mM, 3 μl), VP buffer (50mM Tris, 0.1% Triton-X100, pH 7.6, 3 μl) and **p53CD_GST** stock solution (7000 ng/μl in 25mM Hepes pH 7.6, 200mM KCl, 10% glycerol, 1mM DTT, 1mM benzamidine; 1.5 μL) in total volume 30 μL. Negative sample of **DNA^{FL}** in buffer was prepared using p53 storage buffer (25mM Hepes pH 7.6, 200mM KCl, 10% glycerol, 1mM DTT, 1mM benzamidine; 1.5 μL) instead of p53 stock solution. Samples

were incubated on ice for 15–30 min. Background-corrected fluorescence spectra were recorded at 5 °C using a 20 µL quartz cuvette (λ_{ex} = 370 nm).

Binding of DNA^{FL} to DOTAP

Titration of **DNA^{FL}** by DOTAP was performed in a 100 µL quartz cuvette at room temperature (22–23 °C). Initial solution containing **DNA^{FL}** (0.5 µM in 10 mM sodium phosphate buffer pH = 7.4, 200 mM NaCl) was titrated by DOTAP liposomes (1 mM in the same buffer). Aliquots of DOTAP (1µL) were added, the solution was carefully mixed with a pipette and equilibrated for 2 minutes before the fluorescence spectrum was recorded (λ_{ex} = 370 nm). Measurements were triplicated. Spectral center of mass (SCM, cm^{-1}) was plotted as a function of DNA/DOTAP charge ratio.

F. Computational Details

General methods

All the calculations were performed using Gaussian09 and Turbomole 7.0 program packages.[S8] The calculations of the absorption and emission spectra were performed using time-dependent density functional theory methods - TD-DFT[S9,S10] and employing the hybrid functional B3-LYP.[S11] To account for a proper description of the excited states with charge transfer character resolution-of-identity algebraic diagrammatic construction through second order method with the resolution of identity method (RI-ADC(2)) [S12-14] employing def2-TZVP basis set. The ground state geometry was optimized by the standard DFT method employing B3-LYP functional, whereas the excited state (S_1) minima were obtained with TD-DFT/B3LYP/def2-SVP method. The effect of the solvent on the emission and absorption energies were studied at the ADC(2) level by means of conductor-like screening model (COSMO) theory as implemented in Turbomole 7.0.

Molecular modeling

The complex of p53 with DNA (PDBID 3EXJ) was modified in the YASARA modelling package.[S15] Fluorene molecules were bound to DNA, H atoms were added to the protein to mimic neutral pH and their positions were optimized. The glycerol and water molecules were removed from the model. The parameter set used for the protein was AMBER ff03.[S16] The ligand (Fluorene) was optimized in a vacuum and partial charges on its atoms were obtained by a restrained fit to the electrostatic potential (RESP) at the AM1BCC level.[S17] Whole complex was minimised and later on anealed by YASARA protocols.[S15]

G. TDFS measurements

Instrumentation

Stationary emission spectra were obtained on Fluorolog-3 spectrofluorometer (model FL3-11; HORIBA Jobin Yvon) equipped with a 450W Xenon-arc lamp. All spectra were collected in 1 nm steps (2 nm bandwidths were chosen for both the excitation and emission monochromators). Emission spectra were measured at magic angle polarization.

Time-resolved fluorescence decays were measured using the time-correlated single photon counting (TCSPC) technique on an IBH 5000 U SPC instrument equipped with a cooled Hamamatsu R3809U-50 microchannel plate photomultiplier with 40 ps time resolution and time setting of 7 ps per channel. Bandwidths for both the excitation and emission monochromators were set to 32 or 16 nm. In order to eliminate scattered light, a 399 nm cut-off filter was used. Samples were excited at 375 nm with an IBH NanoLED-11 diode laser (70 ps fwhm) with a repetition frequency of 1 MHz. The detected signal was kept below 20 000 counts per second in order to avoid shortening of the recorded lifetime due to the pile-up effect. Fluorescence decays were collected at magic angle polarization and fitted (using the iterative reconvolution procedure with PicoQuant FluoFit® software) to a multiexponential function (eq. 1) convoluted with the experimental response function IRF ("prompt"), yielding sets of lifetimes τ_i and corresponding amplitudes A_i.

$$I(t) = \sum_i A_i e^{-t/\tau_i} \otimes IRF$$

Stationary emission spectra and time-resolved fluorescence decays were collected at room temperature (23 ^{o}C) unless otherwise stated.

Method

We used **dC^{FL}** as a probe to study the polarity and hydration around DNA using time dependent fluorescence shift (TDFS) experiments. TDFS experiments utilize an ultrafast change in the dipole moment caused by electronic excitation of the fluorescence dye. The subsequent time evolution of the fluorescence contains complex information about static and dynamic properties of the microenvironment of the dye. The most common method to extract this information is to analyze the time resolved emission spectra (TRES). As it was shown for a large variety of neat liquids, following temporal changes of TRES spectral maximum $v(t)$ the micropolarity and microviscosity of the dye microenvironment can be assessed.[S18, S19] The micropolarity can be characterized by the total amount of the TDFS which is defined as:

$$\Delta v = v(0) - v(\infty)$$

Using the normalization of the temporal dependence of TRES maxima $v(t)$ to the total amount of the TDFS one can characterize the kinetics of the relaxation process:

$$C(t) = \frac{v(t) - v(\infty)}{\Delta v}$$

$C(t)$, also known as *spectral response function*, reflects the rearrangement kinetics of the immediate vicinity of the fluorophore. Thus, the magnitude and the temporal evolution of the TDFS should reflect the dynamics of the hydration shell in the vicinity of **dC^{FL}** exposed to the major groove of DNA. Average relaxation time

τ_R is proportional to the microviscosity or mobility of the dye microenvironment and it can be characterized by the integration of the $C(t)$ function over time:[S19]

$$\tau_R = \int_0^{\infty} C(t)dt$$

TRES points $C(\lambda, t)$ are calculated by a relative normalization of the fitted fluorescence decays $I(\lambda,t)$ to the steady state spectrum $S_0(\lambda)$ at a given time:

$$S(\lambda, t) = \frac{I(\lambda, t)S_0(\lambda)}{\int_0^{\infty} I(\lambda, t)dt}$$

The evaluation of the dynamic Stokes shift is obviously limited by the temporal resolution of the lifetime measurements.

To reveal if the relaxation process or a part of this process is faster than the resolution limit of the measuring device, we used the Fee-Maroncelli procedure to obtain the percentage of the solvation that is missed.[S20] When the excitation is near the maximum of the absorption and individual vibrational modes are not resolved (the absorption and emission spectra are unstructured), then the estimation of the position of the emission maxima at "time zero" $v_{em}^{p}(0)$ can be simplified as follows:

$$v_{em}^{p}(0) \approx v_{abs}^{p} - (v_{abs}^{np} - v_{em}^{np})$$

Where $v_{abs}^{p}, v_{abs}^{np}$ and v_{em}^{np} are the absorption frequency in polar medium, absorption and emission frequencies in nonpolar medium, respectively. To estimate the position frequencies we used midpoint frequency:

$$v_{md} = \frac{1}{2}(v_- + v_+)$$

Where v_- and v_+ are the frequencies on the low and high frequency sides at the half-maximum value of the appropriate spectra, respectively.[S20]

Sample preparation and measurements

DNA^{FL} in buffer:Mixture contained **DNA^{FL}** (1μM, 3.7μL), KCl (500mM, 2 μl), DTT (2mM, 2 μl), VP buffer (50mM Tris, 0.1% Triton-X100, pH 7.6, 2 μl) and p53 storage buffer (25mM Hepes pH 7.6, 200mM KCl, 10% glycerol, 1mM DTT, 1mM benzamidine; 1.5 μL) in total volume 20 μL. The TCSPC measurements were performed at 5°C using a 20 μL quartz cuvette.

DNA^{FL} in buffer–glycerol:Mixture contained **DNA^{FL}** (1μM, 3.7μL), KCl (500mM, 2 μl), DTT (2mM, 2 μl), VP buffer (50mM Tris, 0.1% Triton-X100, pH 7.6, 2 μl) and glycerol 9.9 μL) in total volume 20 μL. The TCSPC measurements were performed at 5°C using a 20 μL quartz cuvette.

DNA^{FL}–p53CD_GST:Mixture contained **DNA^{FL}** (1μM, 3.7μL), KCl (500mM, 2 μl), DTT (2mM, 2 μl), VP buffer (50mM Tris, 0.1% Triton-X100, pH 7.6, 2 μl) and **p53CD_GST** stock solution (700 ng/μl in 25mM Hepes pH 7.6, 200mM KCl, 10% glycerol, 1mM DTT, 1mM benzamidine; 1.5 μL) in total volume

20 μL. Solution was incubated on ice for 20 min; the TCSPC measurements were performed at 5°C using a 20 μL quartz cuvette.

DNA^{FL}–DOTAP: Solutions of **DNA^{FL}** (0.5 μM) and DOTAP (either 50 μM or 200 μM) in 10 mM sodium phosphate buffer pH 7.4, 200 mM NaCl were prepared with total volume 60 μL. The TCSPC measurements were performed at 23°C using a 60 μL quartz cuvette.

H. Image analysis

Image acquisition and processing

Images of UVA-illuminated solutions were taken in a dark room using digital cameras (Nikon D3000 or Nikon D5100) equipped with an Industar 61 L/D objective. Shutter speed and other parameters were chosen automatically by the camera and varied from frame to frame. The images were saved as JPEG files. The analysis of regions of interest (144×144 pixels) was performed using ImageJ software as described in the literature (ref. 31c in the main text).

Compound 8 in 1,4-dioxane/water mixtures

The color response of the fluorophore was analyzed using digital image of solutions of compound **8** in dioxane-water mixtures (Figure S1a). Figure S6 shows gradual change of the color, hue (H) and red/green (R/G) ratio segments as a function of solvent mixture composition. Further analysis of the H and R/G values revealed that the dependence was nearly linear (Figure S8). One can also note, that the value of the blue channel is also continuously changing without oscillation or interruption upon the changing amount of water in dioxane whereas the red and green channel together with saturation and brightness are either constant or oscillating (Figure S7).

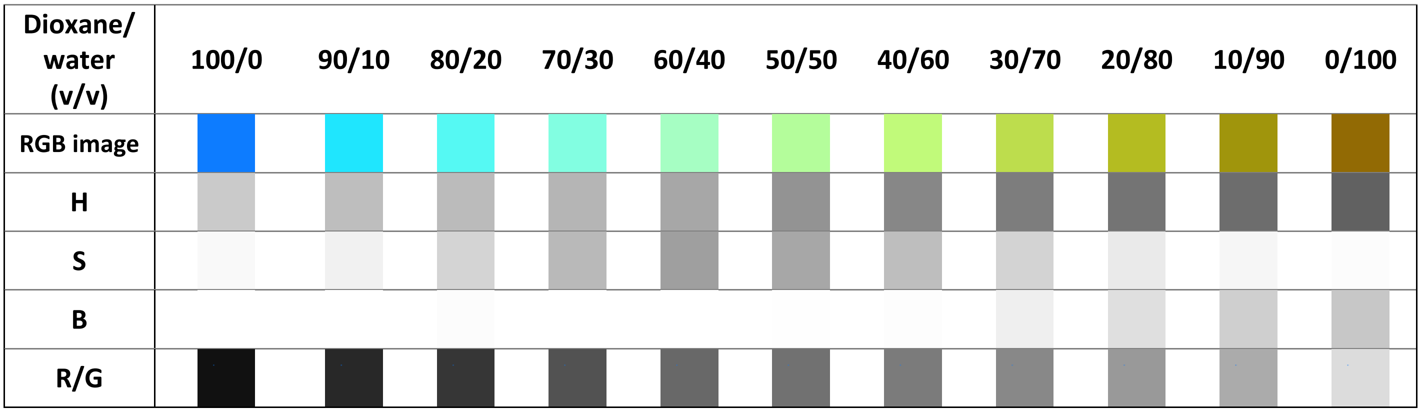

Figure S6. Change of the color of RGB segments, hue (H), saturation (S), brightness (B), red/green ratio (R/G) images with the different dioxane/water ratio (from top to the bottom row**).**

(a) **(b)**

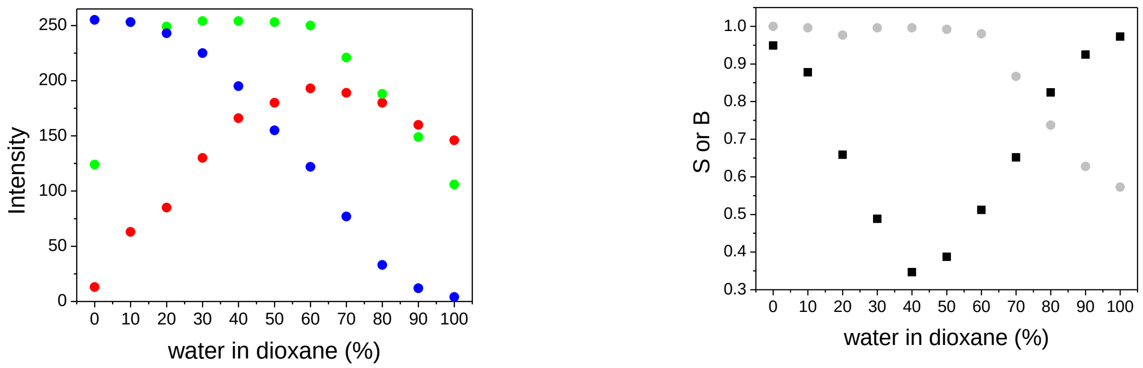

Figure S7. Change of the intensity of red, green and blue channel of the RGB image (a) and change of the saturation (S) (black squares) and brightness (B) (grey circles) (b) upon the addition of water to dioxane

(a) **(b)**

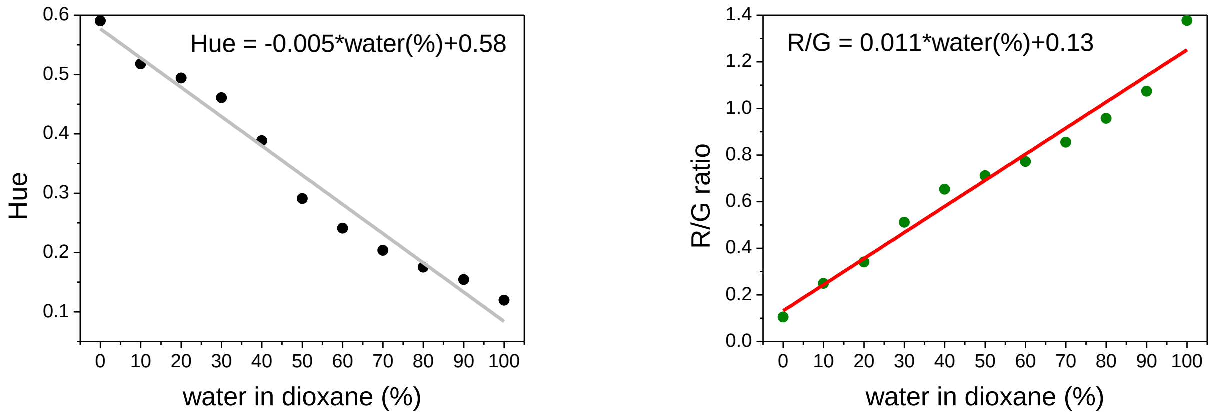

Figure S8. Linear change of the hue (a) and R/G ratio (b) upon the addition of water to dioxane

I. References

S1. A. L. Okorokov and E. V. Orlova, *Curr. Opin. Struct. Biol.*, 2009, **19**, 197–202.

S2. F. Albericio, M. Cruz, L. Debéthune, R. Eritja, E. Giralt, A. Grandas, V. Marchán, J. J. Pastor, E. Pedroso, F. Rabanal and M. Royo, *Synth. Commun.,* 2001, **31**, 225–232.

S3. W. A. Kibbe, *Nucleic Acids Res.*, 2007, **35**, W43–W46.

S4. a) C. Klein, G. Georges, K.-P. Künkele, R. Huber, R. A. Engh and S. Hansen, *J. Biol. Chem.*, 2001, **276**, 37390–37401; b) J. Buzek, L. Latonen, S. Kurki, K. Peltonen and M. Laiho, *Nucleic Acids Res.*, 2002, **30**, 2340–2348; c) M. Fojta, H. Pivonkova, M. Brazdova, K. Nemcova, J. Palecek and B. Vojtesek, *Eur. J. Biochem.*, 2004, **271**, 3865–3876; d) M. Brázdová, L. Navrátilová, V. Tichý, K. Němcová, M. Lexa, R. Hrstka, P. Pečinka, M. Adámik, B. Vojtesek, E. Paleček, W. Deppert and M. Fojta, *PLoS ONE*, 2013, **8**, e59567.

S5. A. M. Brouwer, *Pure Appl. Chem.*, 2011, **83**, 2213–2228.

S6. C. Würth, M. Grabolle, J. Pauli, M. Spieles and U. Resch-Genger, *Nature Protocols*, 2013, **8**, 1535–1550.

S7. W. H. Melhuish, *Phys. Chem.*, 1961, **65**, 229–235.

S8. M. J. Frisch, G. W. Trucks, H. B. Schlegel, G. E. Scuseria, M. A. Robb, J. R. Cheeseman, G. Scalmani, V. Barone, B. Mennucci, G. A. Petersson, H. Nakatsuji, M. Caricato, X. Li, H. P. Hratchian, A. F. Izmaylov, J. Bloino, G. Zheng, J. L. Sonnenberg, M. Hada, M. Ehara, K. Toyota, R. Fukuda, J. Hasegawa, M. Ishida, T. Nakajima, Y. Honda, O. Kitao, H. Nakai, T. Vreven, J. A. Montgomery, Jr., J. E. Peralta, F. Ogliaro, M. Bearpark, J. J. Heyd, E. Brothers, K. N. Kudin, V. N. Staroverov, R. Kobayashi, J. Normand, K. Raghavachari, A. Rendell, J. C. Burant, S. S. Iyengar, J. Tomasi, M. Cossi, N. Rega, J. M. Millam, M. Klene, J. E. Knox, J. B. Cross, V. Bakken, C. Adamo, J. Jaramillo, R. Gomperts, R. E. Stratmann, O. Yazyev, A. J. Austin, R. Cammi, C. Pomelli, J. W. Ochterski, R. L. Martin, K. Morokuma, V. G. Zakrzewski, G. A. Voth, P. Salvador, J. J. Dannenberg, S. Dapprich, A. D. Daniels, Ö. Farkas, J. B. Foresman, J. V. Ortiz, J. Cioslowski and D. J. Fox, GAUSSIAN 09 (Revision A.1.), Gaussian, Inc., Wallingford CT, 2009.

S9. R. Bauerschmitt and R. Ahlrichs, *Chem. Phys. Lett.*, 1996, **256**, 454-464.

S10. E. Runge and E. K. U. Gross, *Phys. Rev. Lett.*, 1984, **52**, 997-1000.

S11. A. D. Becke, *J. Chem. Phys.*, 1993, **98**, 5648-5652.

S12. A. B. Trofimov and J. Schirmer, *J. Phys. B: At. Mol. Opt. Phys.*, 1995, **28**, 2299-2324.

S13. C. Hättig *Phys. Chem. Chem. Phys.*, 2005, **7**, 59-66.

S14. A. B. Trofimov and J. Schirmer, *Chem. Phys.*, 1997, **214**, 153-170.

S15. E. Krieger, K. Joo, J. Lee, S. Raman, J. Thompson, M. Tyka, D. Baker and K. Karplus, *Proteins*, 2009, **77(Suppl 9)**, 114-122.

S16. Y. Duan, C. Wu, S. Chowdhury, M. C. Lee, G. Xiong, W. Zhang, R. Yang, P. Cieplak, R. Luo, T. Lee, J. Caldwell, J. Wang and P. Kollman, *J. Comput. Chem.*, 2003, **24**, 1999-2012.

S17. A. Jakalian, D. B. Jack and C. I. Bayly, *J. Comput. Chem.*, 2002, **23**, 1623-1641.

S18. P. Jurkiewicz, L. Cwiklik, P. Jungwirth and M. Hof, *Biochimie*, 2012, **94**, 26–32.

S19. P. Jurkiewicz, J. Sykora, A. Olzynska, J. Humpolickova and M. Hof, *Journal of Fluorescence*, 2005, **15**, 883–894.

S20. R. Fee and M. Maroncelli, *Chemical Physics*, 1994, **183**, 235–247.

J. Copies of NMR spectra

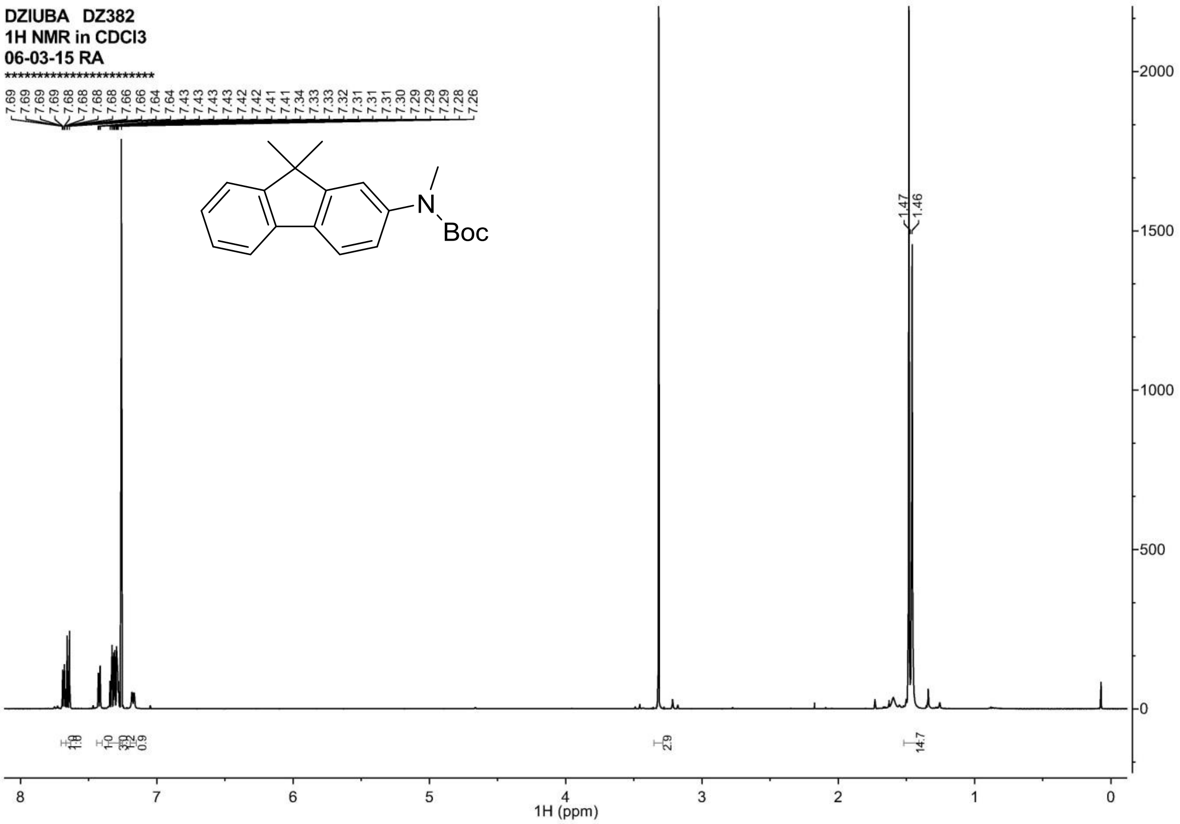

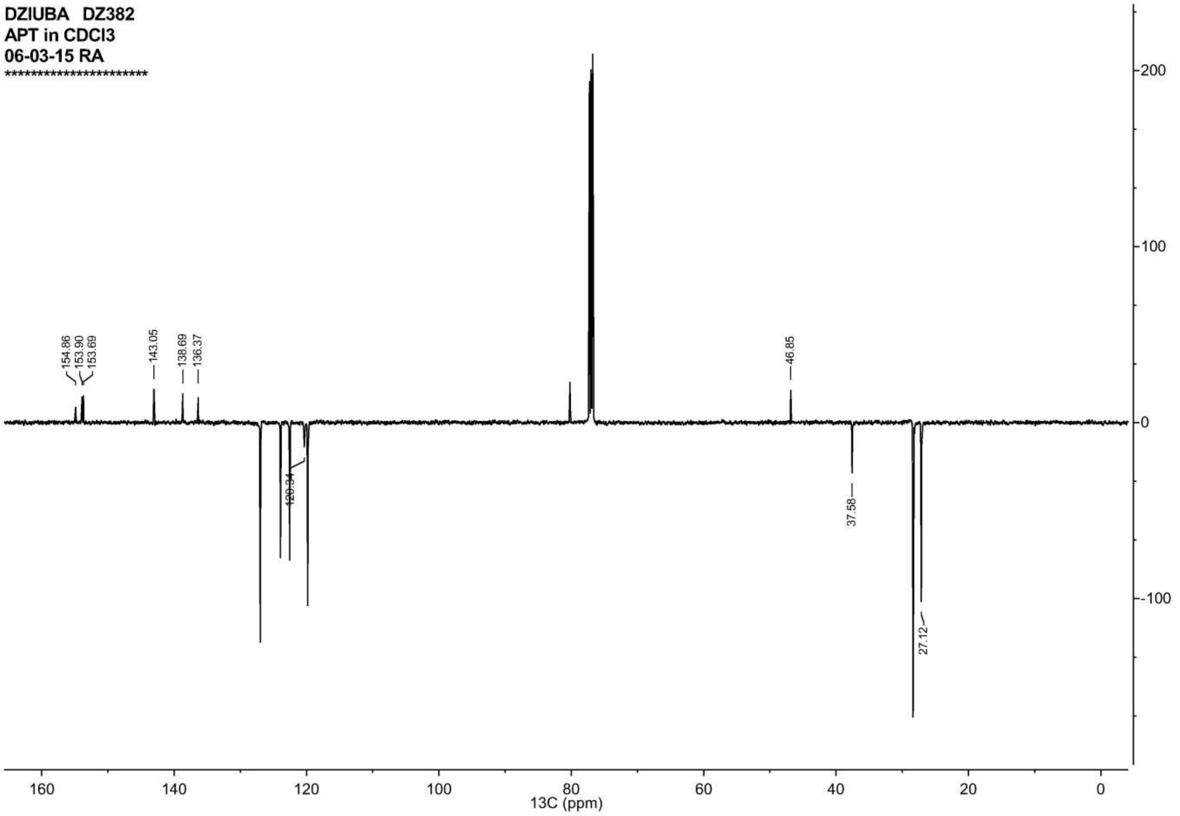

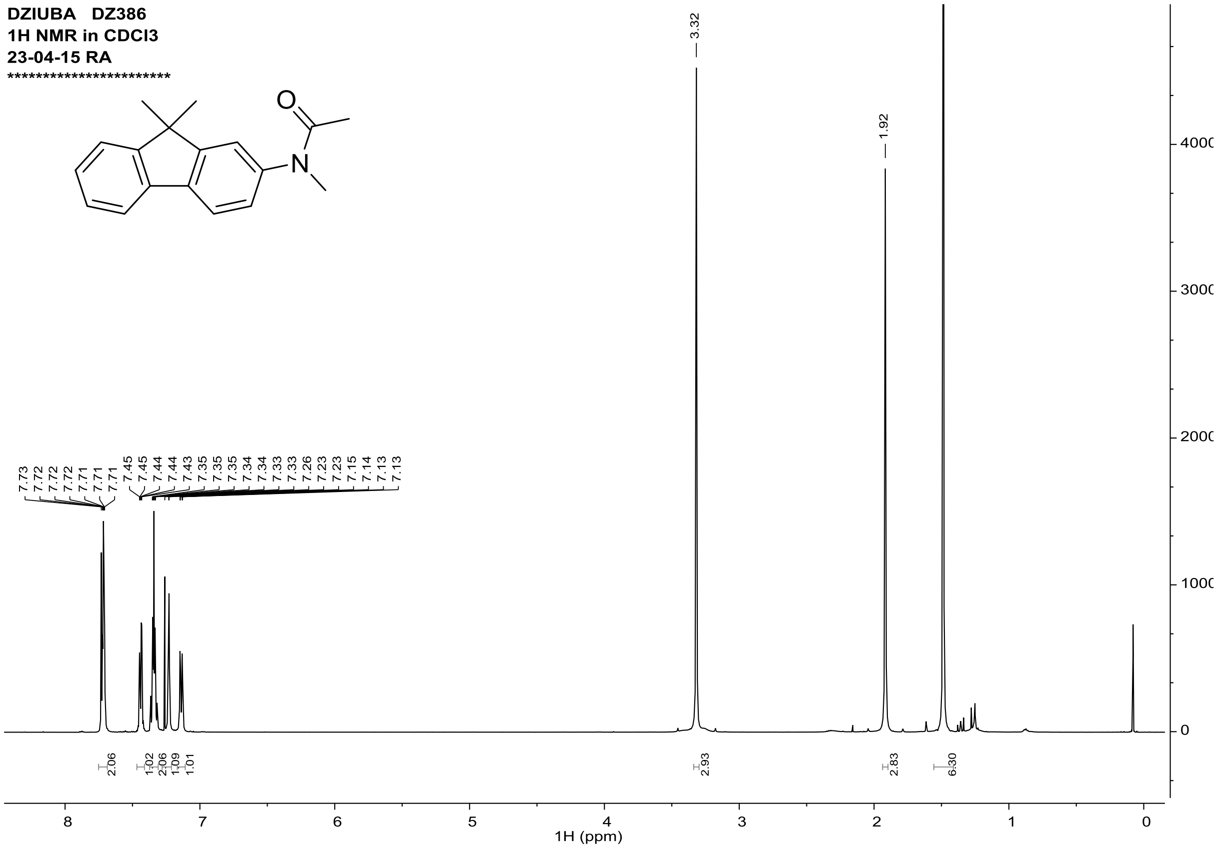
DZIUBA DZ386
1H NMR in CDCl3
23-04-15 RA
1H (ppm)

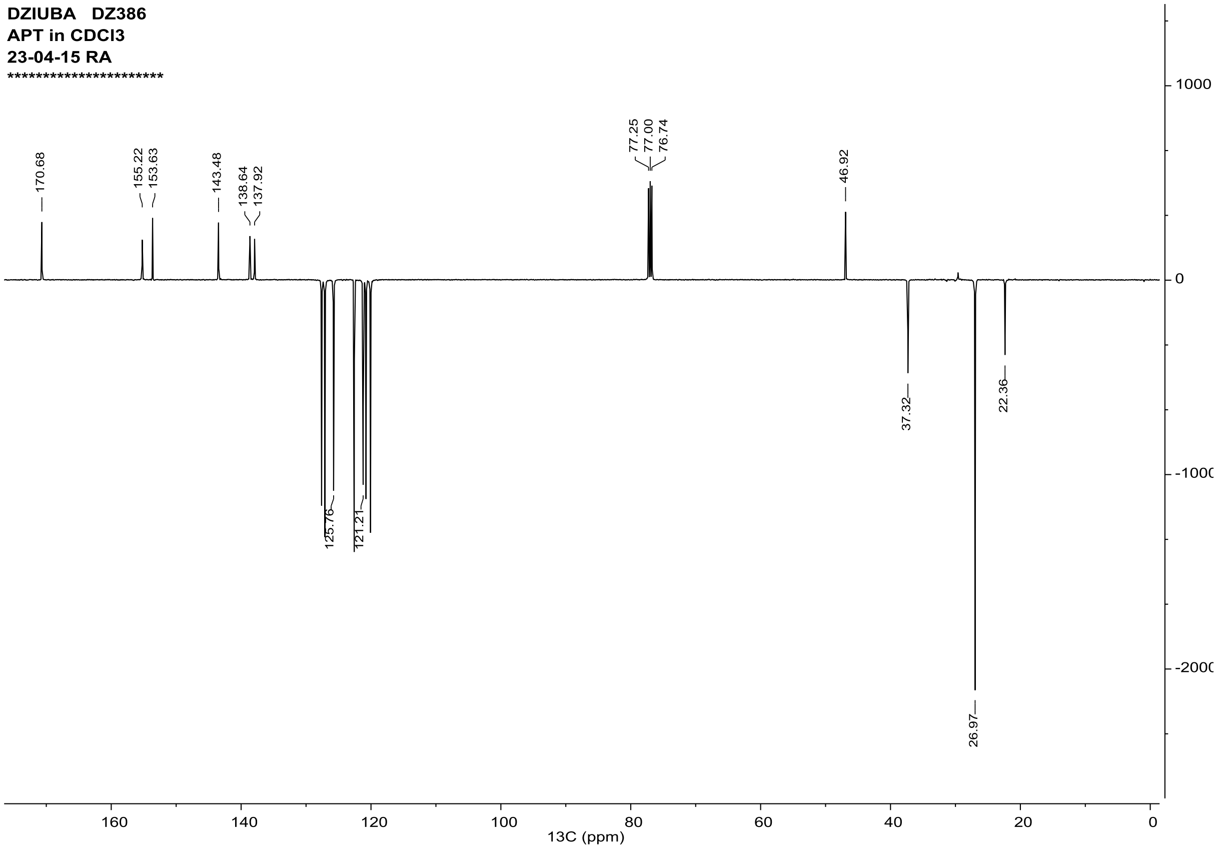
DZIUBA DZ386
APT in CDCl3
23-04-15 RA
13C (ppm)

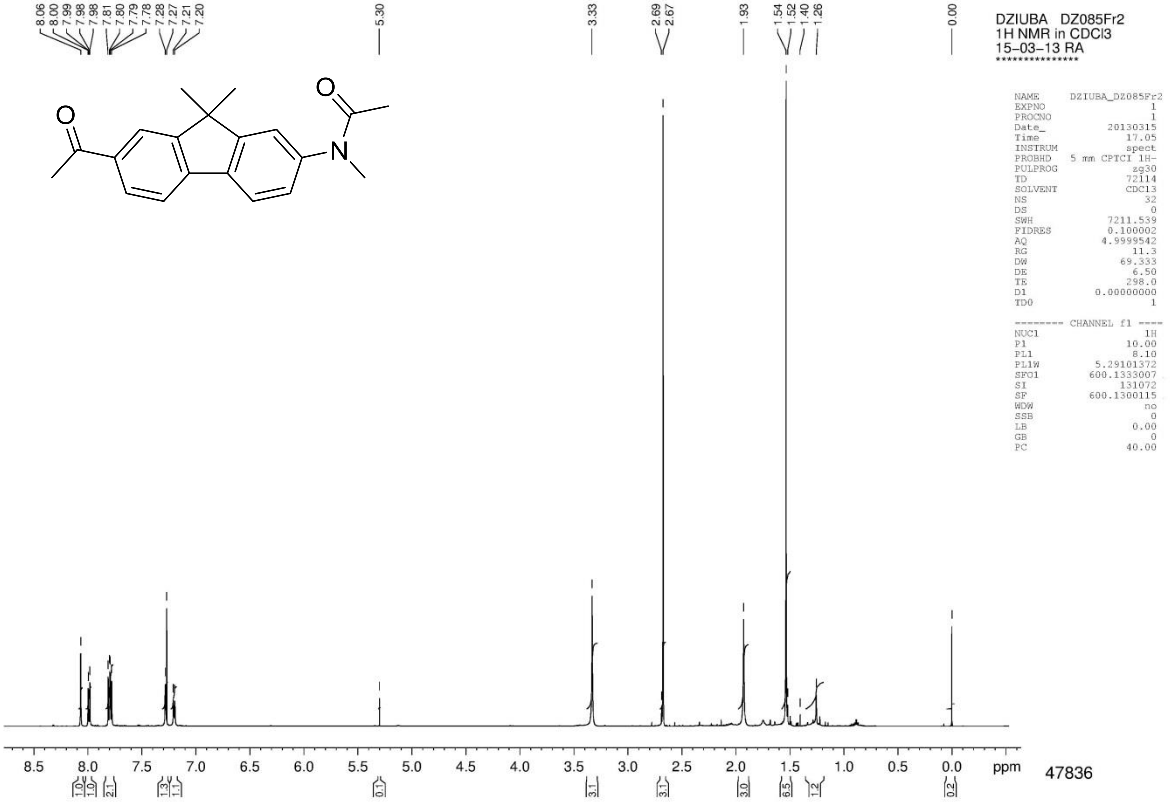

8.06
8.00
7.99
7.98
7.98
7.81
7.80
7.79
7.78
7.28
7.27
7.21
7.20
5.30
3.33
2.69
2.67
1.93
1.54
1.52
1.40
1.26
0.00
DZIUBA DZ085Fr2
1H NMR in CDCl3
15–03–13 RA
NAME DZIUBA_DZ085Fr2
EXPNO 1
PROCNO 1
Date_ 20130315
Time 17.05
INSTRUM spect
PROBHD 5 mm CPTCI 1H-
PULPROG zg30
TD 72114
SOLVENT CDCl3
NS 32
DS 0
SWH 7211.539
FIDRES 0.100002
AQ 4.9999542
RG 11.3
DW 69.333
DE 6.50
TE 298.0
D1 0.00000000
TD0 1
======== CHANNEL f1 ====
NUC1 1H
P1 10.00
PL1 8.10
PL1W 5.29101372
SFO1 600.1333007
SI 131072
SF 600.1300115
WDW no
SSB 0
LB 0.00
GB 0
PC 40.00
8.5 8.0 7.5 7.0 6.5 6.0 5.5 5.0 4.5 4.0 3.5 3.0 2.5 2.0 1.5 1.0 0.5 0.0 ppm
47836
1.0
1.0
2.1
1.3
1.1
0.1
3.1
3.1
3.0
6.5
1.2
0.2

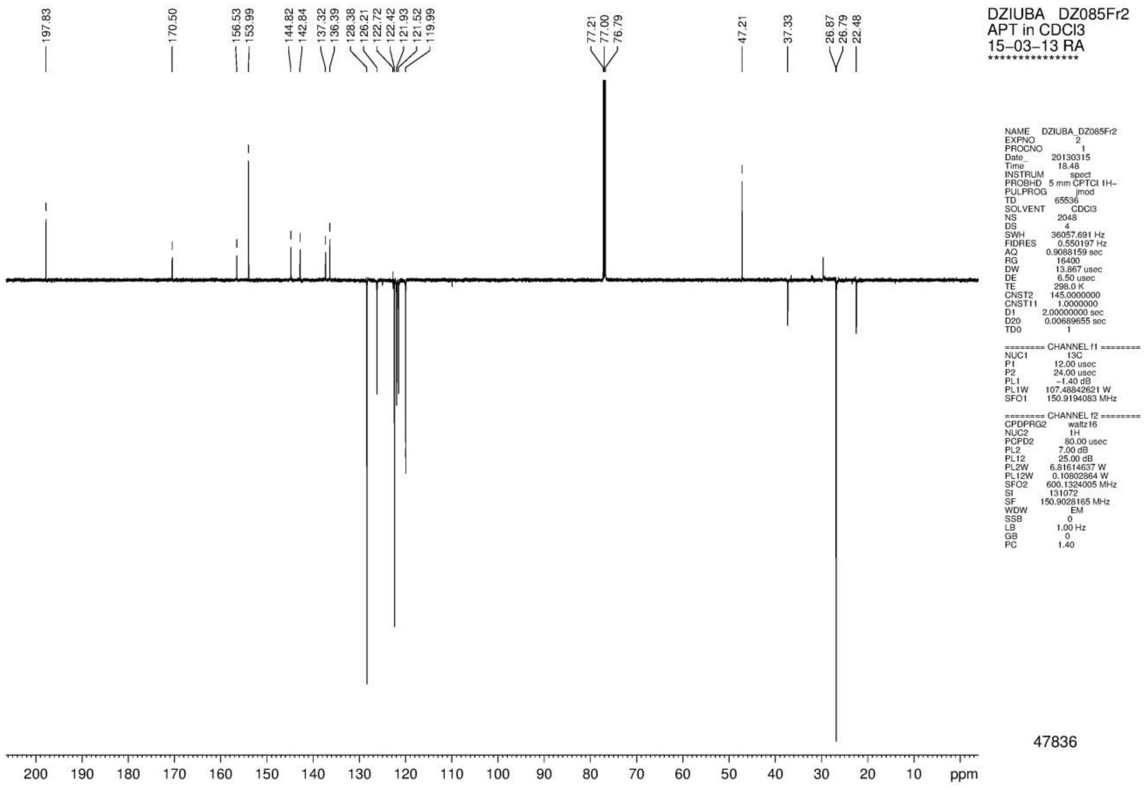

197.83
170.50
156.53
153.99
144.82
142.84
137.32
136.39
128.38
126.21
122.72
122.42
121.93
121.52
119.99
77.21
77.00
76.79
47.21
37.33
26.87
26.79
22.48
DZIUBA DZ085Fr2
APT in CDCl3
15–03–13 RA
NAME DZIUBA_DZ085Fr2
EXPNO 2
PROCNO 1
Date_ 20130315
Time 18.48
INSTRUM spect
PROBHD 5 mm CPTCI 1H-
PULPROG jmod
TD 65536
SOLVENT CDCl3
NS 2048
DS 4
SWH 36057.691 Hz
FIDRES 0.550197 Hz
AQ 0.9088159 sec
RG 16400
DW 13.867 usec
DE 6.50 usec
TE 298.0 K
CNST2 145.0000000
CNST11 1.0000000
D1 2.00000000 sec
D20 0.00689655 sec
TD0 1
======== CHANNEL f1 ========
NUC1 13C
P1 12.00 usec
P2 24.00 usec
PL1 −1.40 dB
PL1W 107.48842621 W
SFO1 150.9194083 MHz
======== CHANNEL f2 ========
CPDPRG2 waltz16
NUC2 1H
PCPD2 80.00 usec
PL2 7.00 dB
PL12 25.00 dB
PL2W 6.81614637 W
PL12W 0.10802864 W
SFO2 600.1324005 MHz
SI 131072
SF 150.9028165 MHz
WDW EM
SSB 0
LB 1.00 Hz
GB 0
PC 1.40
47836
200 190 180 170 160 150 140 130 120 110 100 90 80 70 60 50 40 30 20 10 ppm

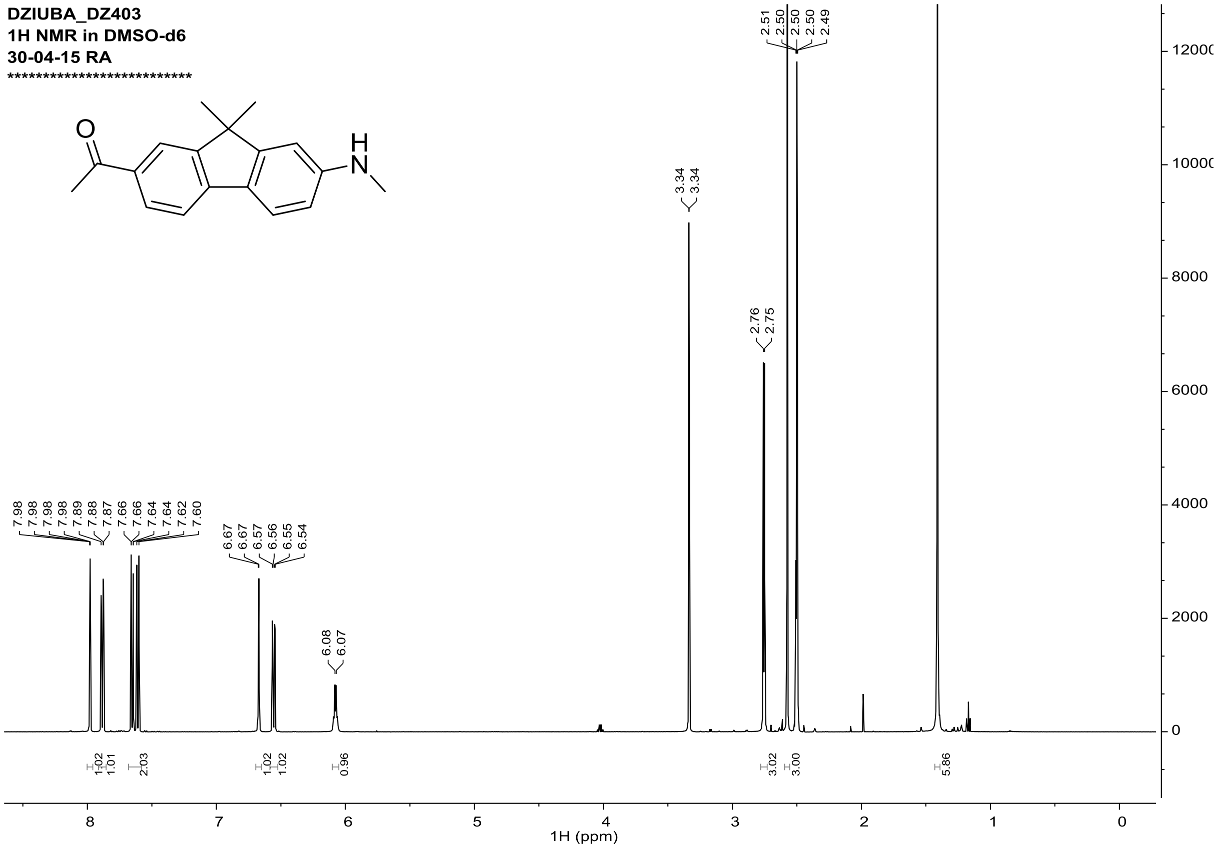
DZIUBA_DZ403
1H NMR in DMSO-d6
30-04-15 RA
****
7.98 7.98 7.98 7.98 7.89 7.88 7.87 7.66 7.66 7.64 7.64 7.62 7.60
6.67 6.67 6.57 6.56 6.55 6.54
6.08 6.07
3.34 3.34
2.76 2.75
2.51 2.50 2.50 2.50 2.49
1.02 1.01 2.03 1.02 1.02 0.96 3.02 3.00 5.86
1H (ppm)

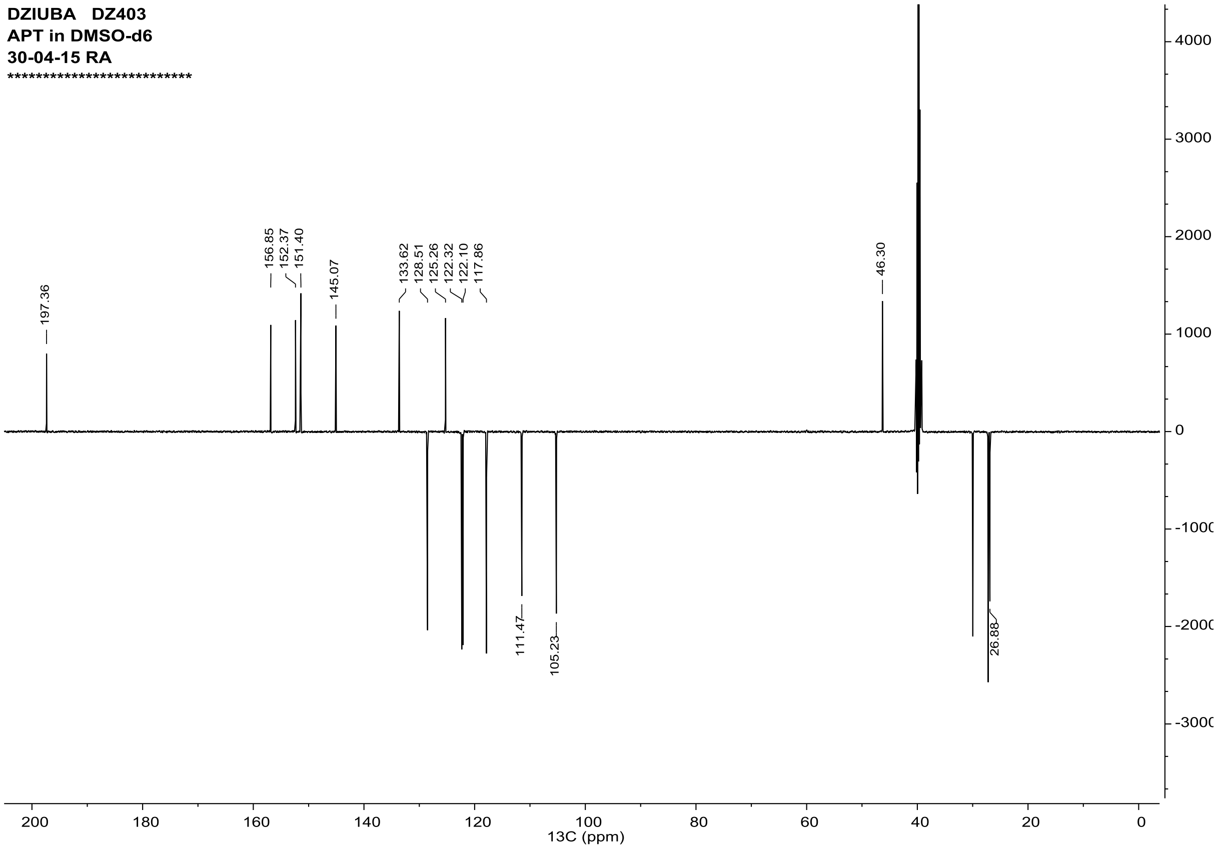
DZIUBA DZ403
APT in DMSO-d6
30-04-15 RA
****
197.36
156.85 152.37 151.40
145.07
133.62 128.51 125.26 122.32 122.10 117.86
111.47
105.23
46.30
26.88
13C (ppm)

DZIUBA DZ418
1H NMR in CDCl3
01-06-15 RA

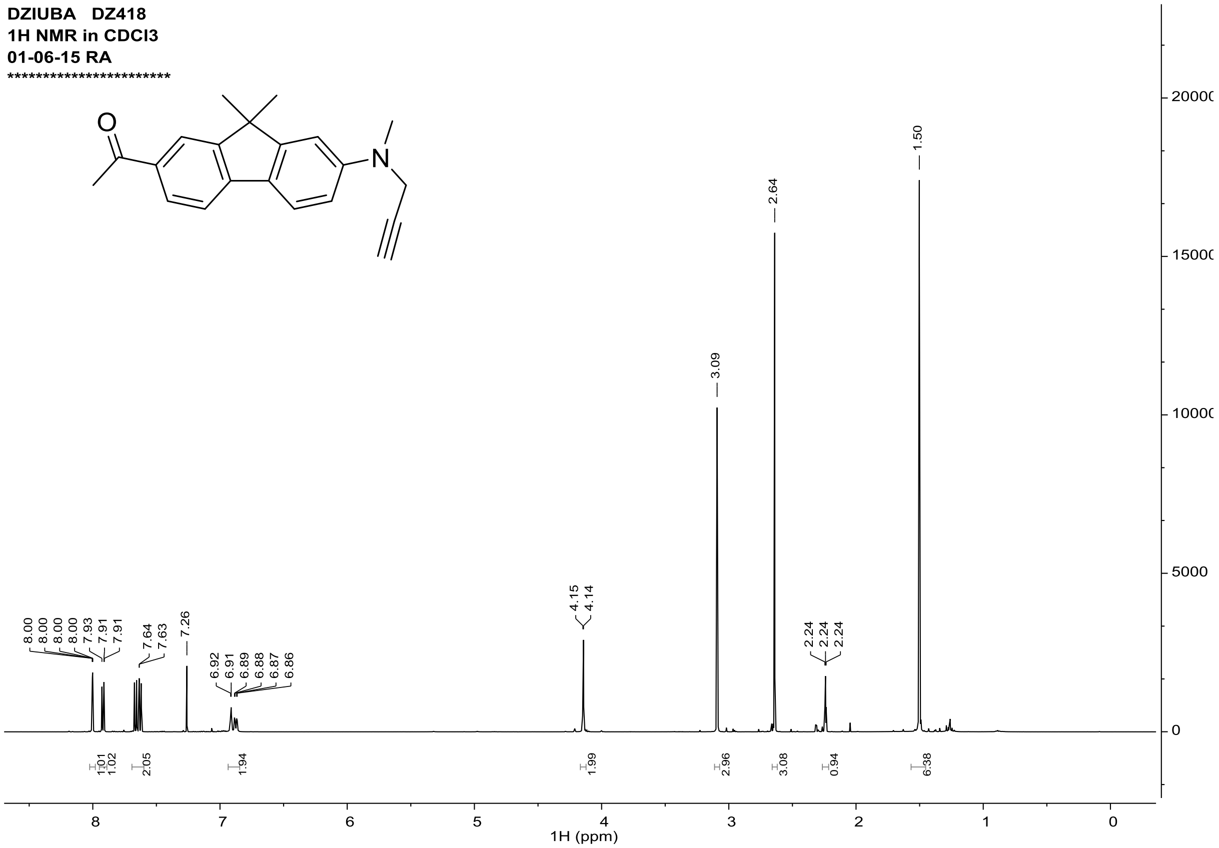

DZIUBA DZ418
APT in CDCl3
01-06-15 RA

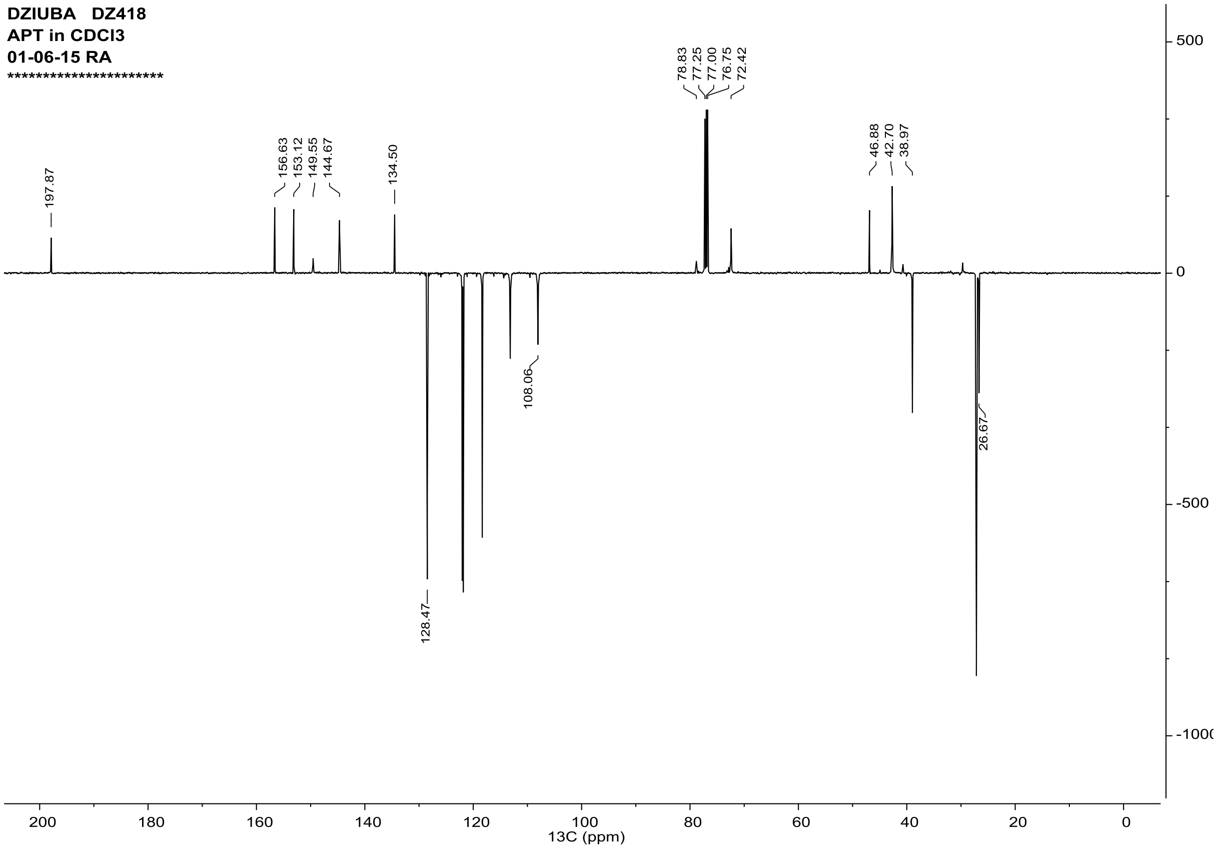

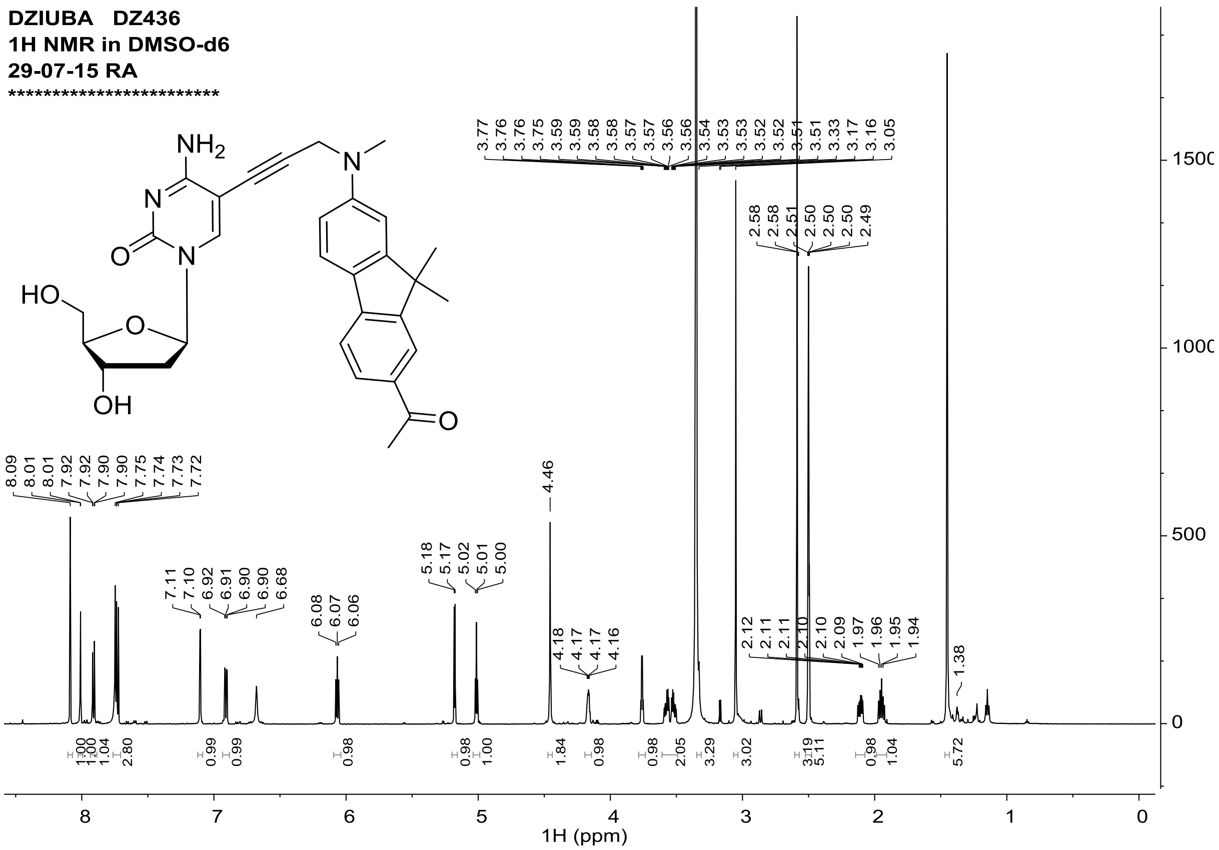
DZIUBA DZ436
1H NMR in DMSO-d6
29-07-15 RA
****
NH2
N
O
N
HO
O
OH
N
O
8.09 8.01 8.01 7.92 7.92 7.90 7.90 7.75 7.74 7.73 7.72
7.11 7.10 6.92 6.91 6.90 6.90 6.68
6.08 6.07 6.06
5.18 5.17 5.02 5.01 5.00
4.46
4.18 4.17 4.17 4.16
3.77 3.76 3.76 3.75 3.59 3.59 3.58 3.58 3.57 3.57 3.56 3.56 3.54 3.53 3.53 3.52 3.52 3.51 3.51 3.33 3.17 3.16 3.05
2.58 2.58 2.51 2.50 2.50 2.50 2.49
2.12 2.11 2.11 2.10 2.10 2.09 1.97 1.96 1.95 1.94
1.38
1.00 1.00 1.04 2.80 0.99 0.99 0.98 0.98 1.00 1.84 0.98 0.98 2.05 3.29 3.02 3.19 5.11 0.98 1.04 5.72
1H (ppm)

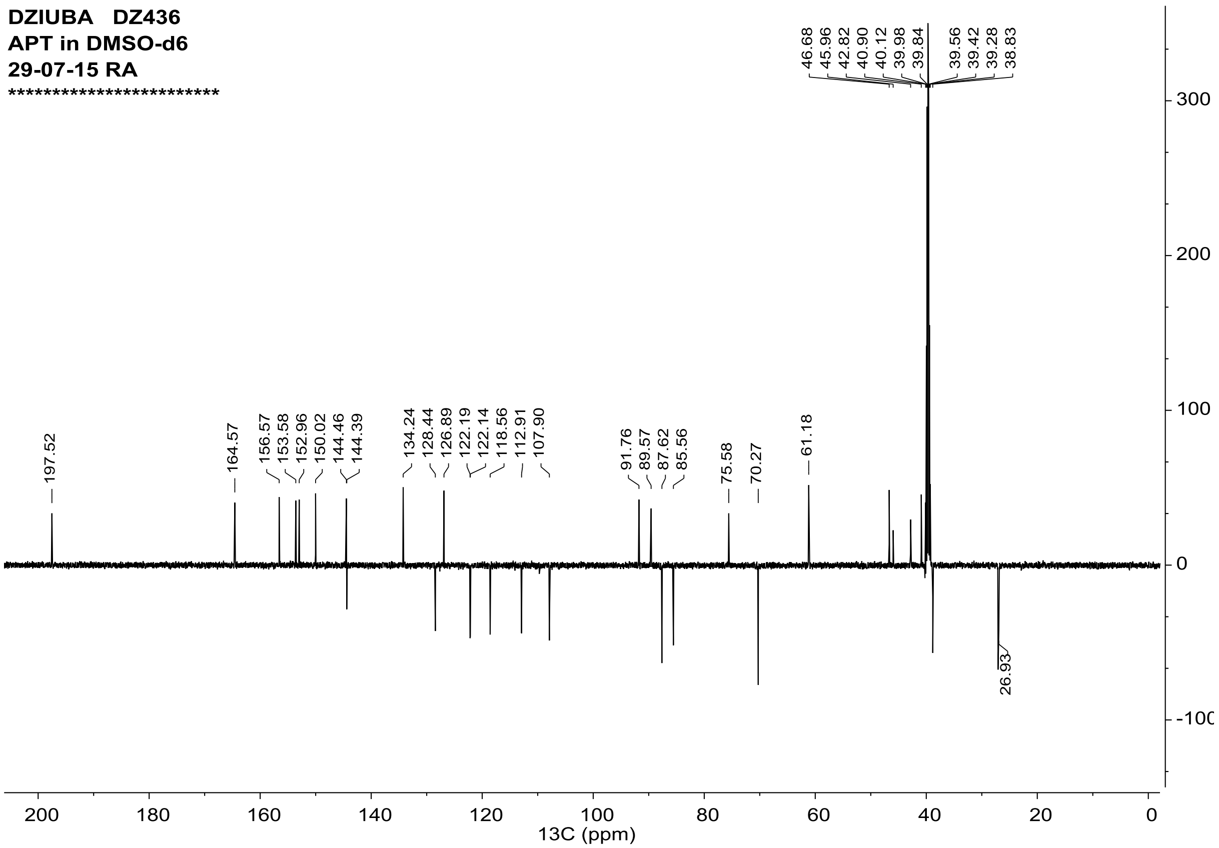
DZIUBA DZ436
APT in DMSO-d6
29-07-15 RA
****
197.52
164.57
156.57 153.58 152.96 150.02 144.46 144.39
134.24 128.44 126.89 122.19 122.14 118.56 112.91 107.90
91.76 89.57 87.62 85.56
75.58
70.27
61.18
46.68 45.96 42.82 40.90 40.12 39.98 39.84 39.56 39.42 39.28 38.83
26.93
13C (ppm)

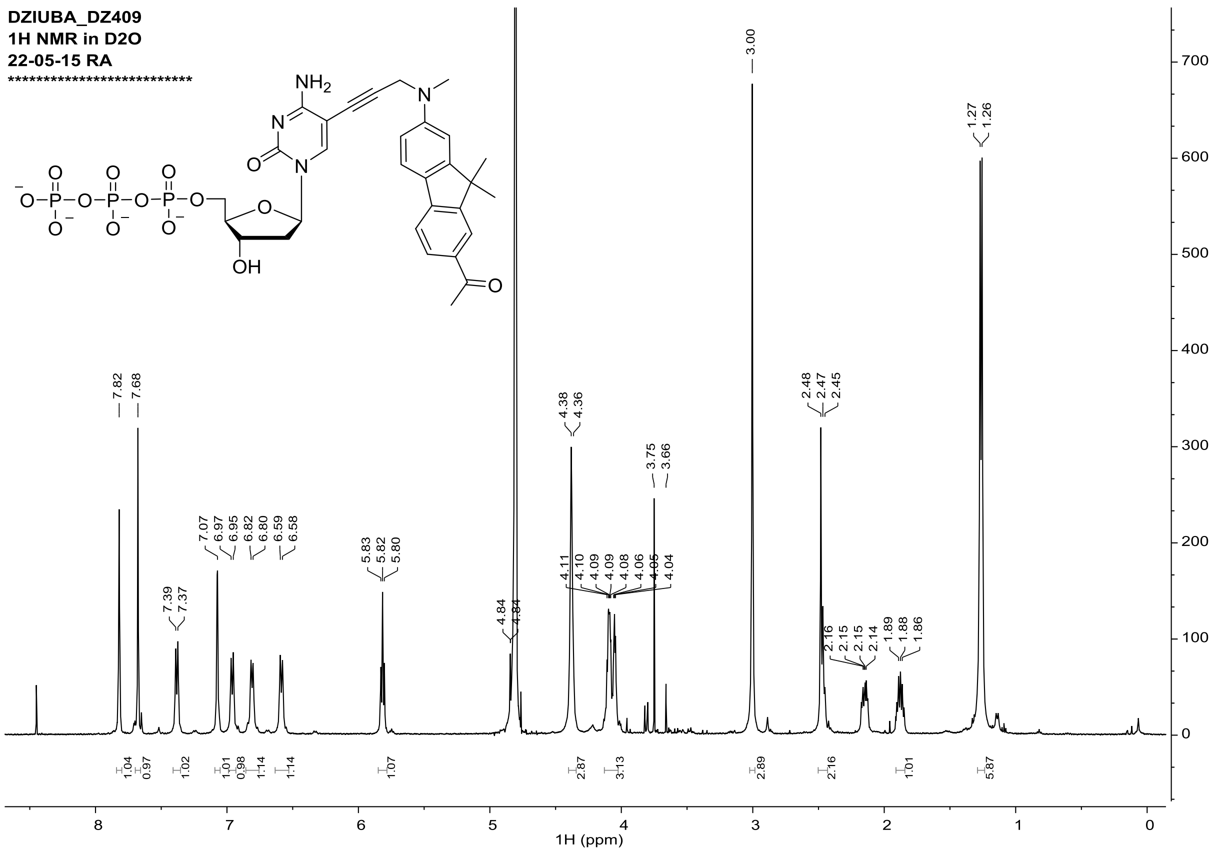
DZIUBA_DZ409
1H NMR in D2O
22-05-15 RA
1H (ppm)

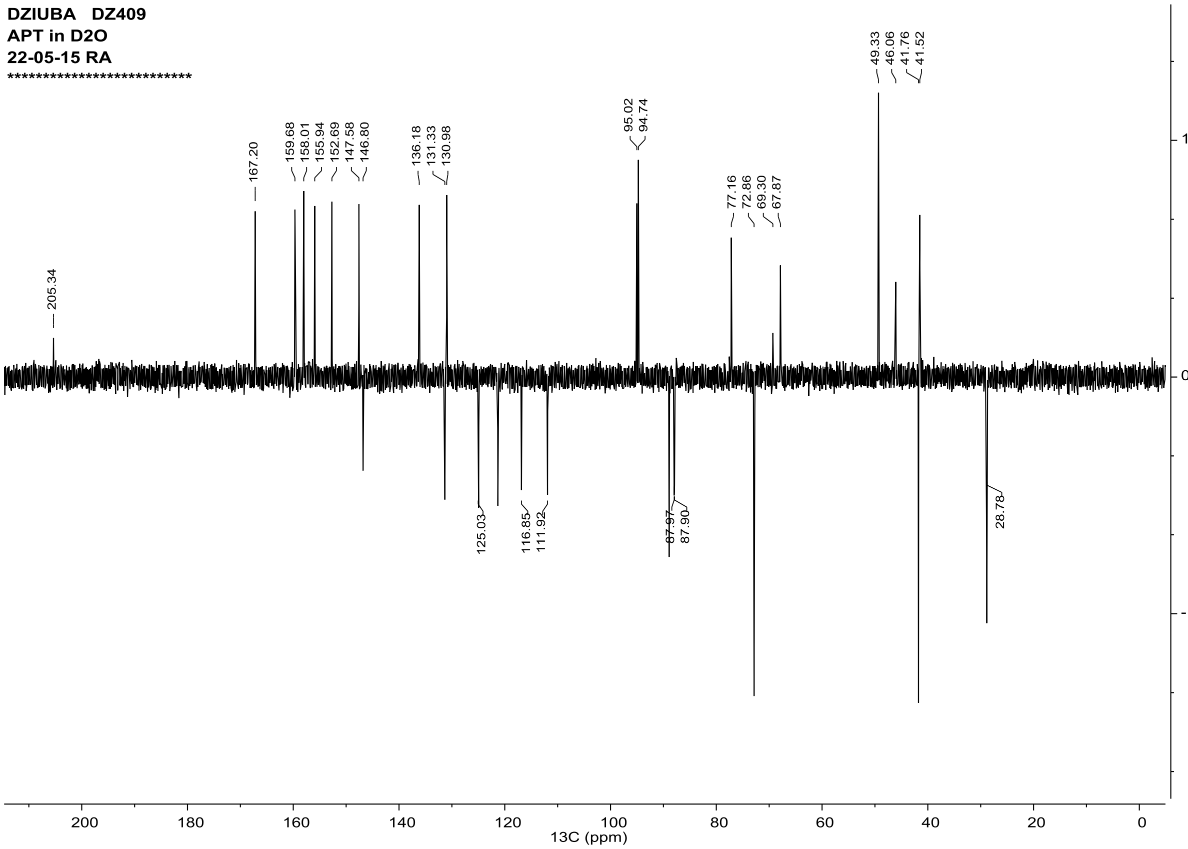
DZIUBA DZ409
APT in D2O
22-05-15 RA
13C (ppm)

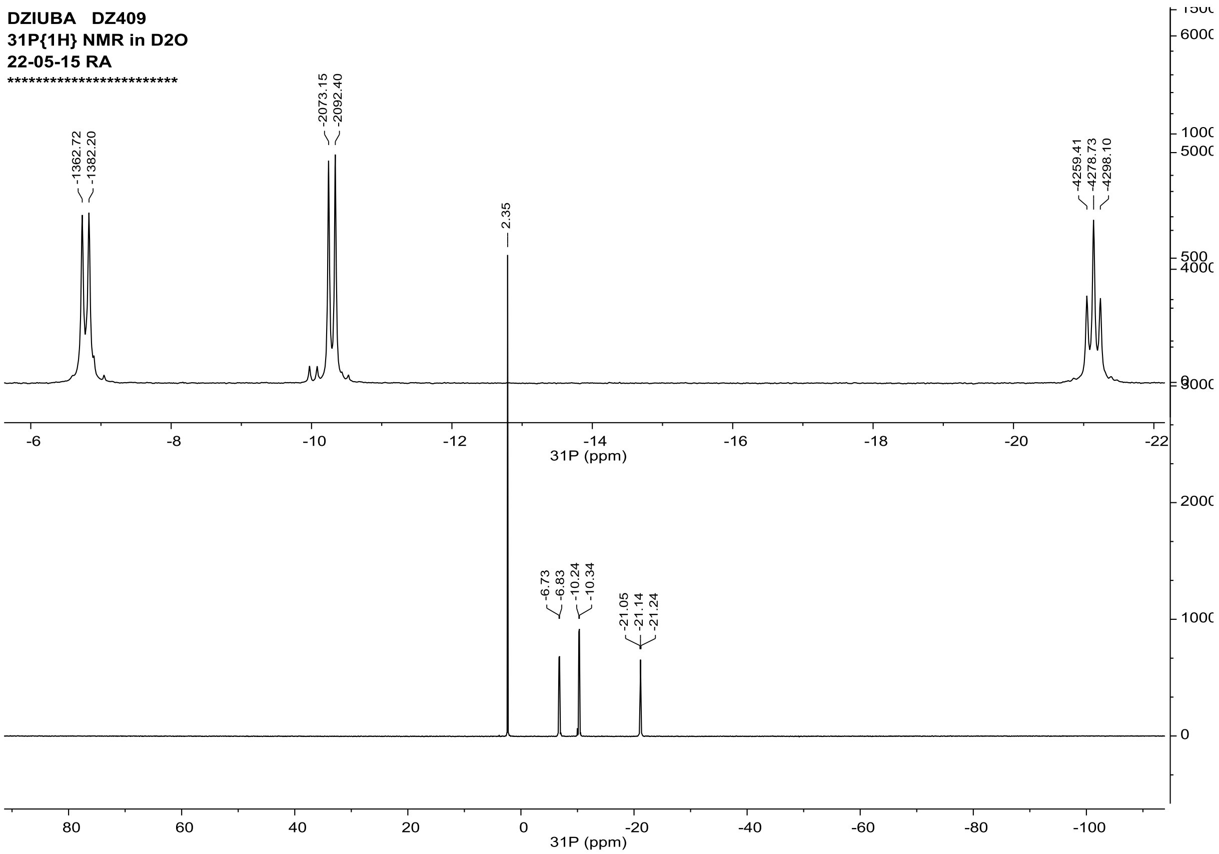
DZIUBA DZ409
31P{1H} NMR in D2O
22-05-15 RA
-1362.72
-1382.20
-2073.15
-2092.40
2.35
-4259.41
-4278.73
-4298.10
31P (ppm)
-6.73
-6.83
-10.24
-10.34
-21.05
-21.14
-21.24
31P (ppm)
